# Regulating Spin Polarization through Topological Defects in Carbon‐Based Metal‐Free Catalyst for Enhanced Fenton‐Like Activity

**DOI:** 10.1002/advs.202514429

**Published:** 2025-08-26

**Authors:** Huajie Zhong, Zeyu Gong, Xi Chen, Bin Zhang, Tao Zhan, Jiaxing Yu, Yu Hou, Yuan Tao, Qi Fu, Huangsheng Yang, Jiating Zheng, Duochu Su, Ganggang Li, Junhui Wang, Gangfeng Ouyang

**Affiliations:** ^1^ School of Chemical Engineering and Technology Sun Yat‐Sen University Zhuhai Guangdong 519082 P. R. China; ^2^ School of Biology and Biological Engineering South China University of Technology Guangzhou Guangdong 510006 P. R. China; ^3^ MOE Key Laboratory of Bioinorganic and Synthetic Chemistry/KLGHEI of Environment and Energy Chemistry School of Chemistry Sun Yat‐Sen University Guangzhou Guangdong 510275 P. R. China; ^4^ Guangzhou Customs Technology Center Guangzhou Guangdong 510623 P. R. China; ^5^ Foshan Fosun Chancheng Hospital Foshan Guangdong 528000 P. R. China; ^6^ National Engineering Laboratory for VOCs pollution Control Material & Technology Research Center for Environmental Material and Pollution Control Technology University of Chinese Academy of Sciences Beijing 101408 P.R. China

**Keywords:** carbocatalysts, carbon defect, Fenton‐like reaction, spin polarization

## Abstract

Regulating spin polarization has been recognized as a promising strategy to improve the catalytic performance across various catalytic domains, since the reaction barriers can be directly influenced by the spin state. However, the existing approaches to modulating the spin polarization of active centers mainly focus on the metal‐based catalysts, and those for the earth‐abundant carbon‐based metal‐free catalysts (CMFCs) are rarely reported. Here, a topological defect engineering strategy is proposed to regulate the spin polarization by introducing pentagon defects on the edge of CMFCs. Theoretical and experimental results indicate that the incorporation of pentagon defects on the edge induces a spin polarization, and more spin‐down electrons locate below the Fermi level, which enhances the electron transfer between peroxymonosulfate (PMS) and the catalyst, and reduces the energy barrier of the key ^*^OOH intermediate during the generation of singlet oxygen (^1^O_2_). As a result, the derived spin‐polarized catalyst (E‐C5) shows remarkable Fenton‐like activity for 4‐chlorophenol (4‐CP) degradation, which is 63 times higher than that of the pristine unpolarized catalyst (E‐C6). This topological defect engineering represents an innovative and effective strategy to regulate spin polarization in carbocatalysts, and provides insightful inspirations for various catalytic fields.

## Graphical abstract

A topological defect engineering strategy is proposed to regulate the spin polarization by introducing pentagon defects on the edge of carbon nanotubes. Theoretical and experimental results indicate that the incorporation of pentagon defects on the edge induces a spin polarization, and more spin‐down electrons locate below the Fermi level, which enhances the generation of singlet oxygen in Fenton‐like reaction.

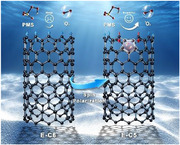

## 1 Introduction

Regulating spin polarization (the uneven proportion of spin‐up and spin‐down electrons at the Fermi energy)^[^ ^1^ ^]^ of catalysts is a promising strategy to boost their catalytic performance in various reactions, like oxygen evolution reaction (OER),^[^ ^2^, ^3^, ^4^, ^5^, ^6^ ^]^ oxygen reduction reaction (ORR),^[^ ^7^, ^8^ ^]^ CO_2_ reduction reaction (CO_2_RR)^[^ ^9^, ^10^, ^11^ ^]^ and advanced oxidation processes.^[^ ^12^ ^]^ For the metal‐based catalysts, manipulating the spin polarization of metal center via regulating the d orbitals’ occupancy (especially the e_g_ orbitals with high energy), which takes part in the σ‐bonding with intermediates and charge transfer at the catalytic interface, plays a crucial role in determining the binding of intermediates to active metal sites and the dynamics of the reaction.^[^ ^13^ ^]^ For example, tailoring the spin polarization of Fe sites fostered a dynamic equilibrium among the activated intermediates and enhanced the balance between the formation of ^*^COOH and the desorption of ^*^CO at the active metal site, thereby an exceptional CO_2_RR activity and selectivity were realized.^[^ ^10^ ^]^ Nevertheless, the high cost, limited availability of precious metals, and negative environmental effect have hindered their large‐scale commercial application.^[^ ^14^ ^]^

To address these challenges, carbon‐based metal‐free catalysts (CMFCs) have been emerging as promising candidates, due to their numerous advantages, such as abundant natural resources, good electrical conductivity, high surface area, excellent thermal stability, and outstanding chemical stability.^[^ ^15^, ^16^, ^17^ ^]^ In order to boost the catalytic performances of CMFCs, doping with heteroatoms on the carbon matrix was proposed to regulate the spin state, such as nitrogen doping^[^ ^18^, ^19^, ^20^, ^21^ ^]^ and sulfur doping.^[^ ^22^, ^23^ ^]^ For instance, the nitrogen doping alters the asymmetry spin density (the difference between the density of the spin‐up electrons and spin‐down electrons),^[^ ^24^ ^]^ rendering it possible for oxygen adsorption.^[^ ^25^ ^]^ Notably, the adjacent C atom bonded to the heteroatoms possesses a high spin density, which is considered the most active site in ORR, and the catalytic performance improves a lot in the presence of defects.^[^ ^26^ ^]^ Indeed, some work suggested that the intrinsic defects could introduce charge into the electron system of the sp^2^‐bonded carbon matrix and tailor the spin densities of carbon atoms.^[^ ^27^, ^28^, ^29^ ^]^ However, the aforementioned strategies mainly focused on the regulation of spin density. The optimization strategies for regulating spin polarization in CMFCs have not been reported yet, and the correlation between spin polarization and the catalytic activity of CMFCs remains indistinct.

Herein, we proposed a topological defect engineering strategy to regulate the spin polarization by introducing pentagon defects on the edge of CMFCs. Importantly, the carbon materials with edged pentagon defects (E‐C5) were prepared from edge defect‐rich model carbon nanotubes (E‐C6) without introducing extra heteroatoms. Peroxymonosulfate (PMS) with asymmetric structure (H─O─O─SO_3_^−^) was chosen as the model superoxide in our study,^[^ ^30^ ^]^ due to the presence of an unbalanced charge distribution within the peroxide O─O bond of PMS, rendering it particularly susceptible to interacting with polarized sites.^[^ ^31^ ^]^ The DFT calculations indicate that the pentagon defects at the edge induce spin polarization, and there are more spin‐down electrons below the Fermi level, disturbing the uniform electron distribution, which enhances the electron transfer between PMS and E‐C5. Furthermore, the energy barrier of the rate‐determining step is reduced in the E‐C5/PMS system, facilitating the generation of singlet oxygen (^1^O_2_). Consequently, the E‐C5/PMS system exhibits a remarkable enhancement in catalytic performance, achieving up to 63 times greater efficiency than the E‐C6/PMS system. This work highlights the controllable manipulation of spin polarization by topological defect engineering to push forward the carbon‐based metal‐free catalysts design and performance improvement.

## 2 Results and Discussion

### 2.1 Pentagon Defects in Engineering on Model Carbocatalysts

**Figure 1:**
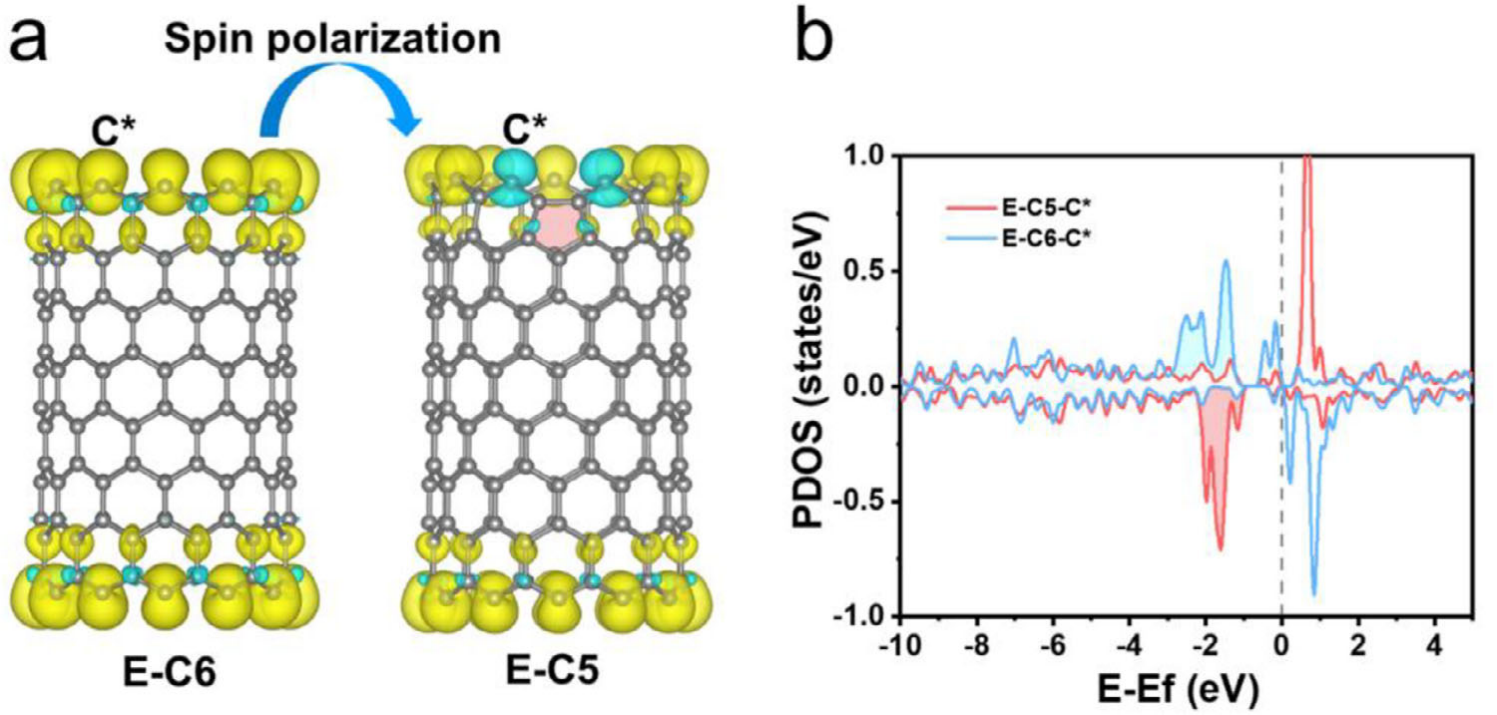
DFT calculations of E‐C5 and E‐C6. a) Spin density distributions of E‐C5 and E‐C6. The cyan and yellow regions represent areas of spin‐up electrons and areas of spin‐down electrons, respectively. b) The partial density of states (PDOS) of E‐C5 and E‐C6.

Pentagon defects were identified as the superior active sites among the intrinsic defects in various catalytic reactions according to the previous works.^[^ ^32^, ^33^ ^]^ Therefore, this kind of defect has been given priority in consideration. First, density functional theory (DFT) calculations were applied to identify the effect of intrinsic pentagon defects on the spin states of a classical carbon material (carbon nanotubes, CNTs). Two theoretical models that correspond to CNTs with common edge defects (E‐C6) and intrinsic pentagon defects at the edge (E‐C5) (Figures S1 and S2, Supporting Information) were established. Additionally, the value of spin densities was further calculated (Figures S3 and S4, Tables S1 and S2, Supporting Information). As shown in **Figure** **1**a, spin‐up electrons are uniformly distributed at the carbon atoms of edge defects (C6).^[^ ^34^ ^]^ However, upon the introduction of a pentagon defect at the edge, the spin flip occurs, and spin‐down electrons are located at the carbon atoms next to the pentagon defect, resulting in the emergence of spin polarization. This is also validated by the partial density of states (PDOS) (Figure 1b). More spin‐down electrons under the Fermi level are observed, suggesting E‐C5 can provide more spin‐down electrons during reactions.^[^ ^12^ ^]^ The above results indicate that the incorporation of a pentagon defect could effectively induce the spin polarization and disturb the uniform electron distribution along the edge of the CNT, which is anticipated to accelerate the electron flow from the catalysts.^[^ ^35^ ^]^

**Figure 2:**
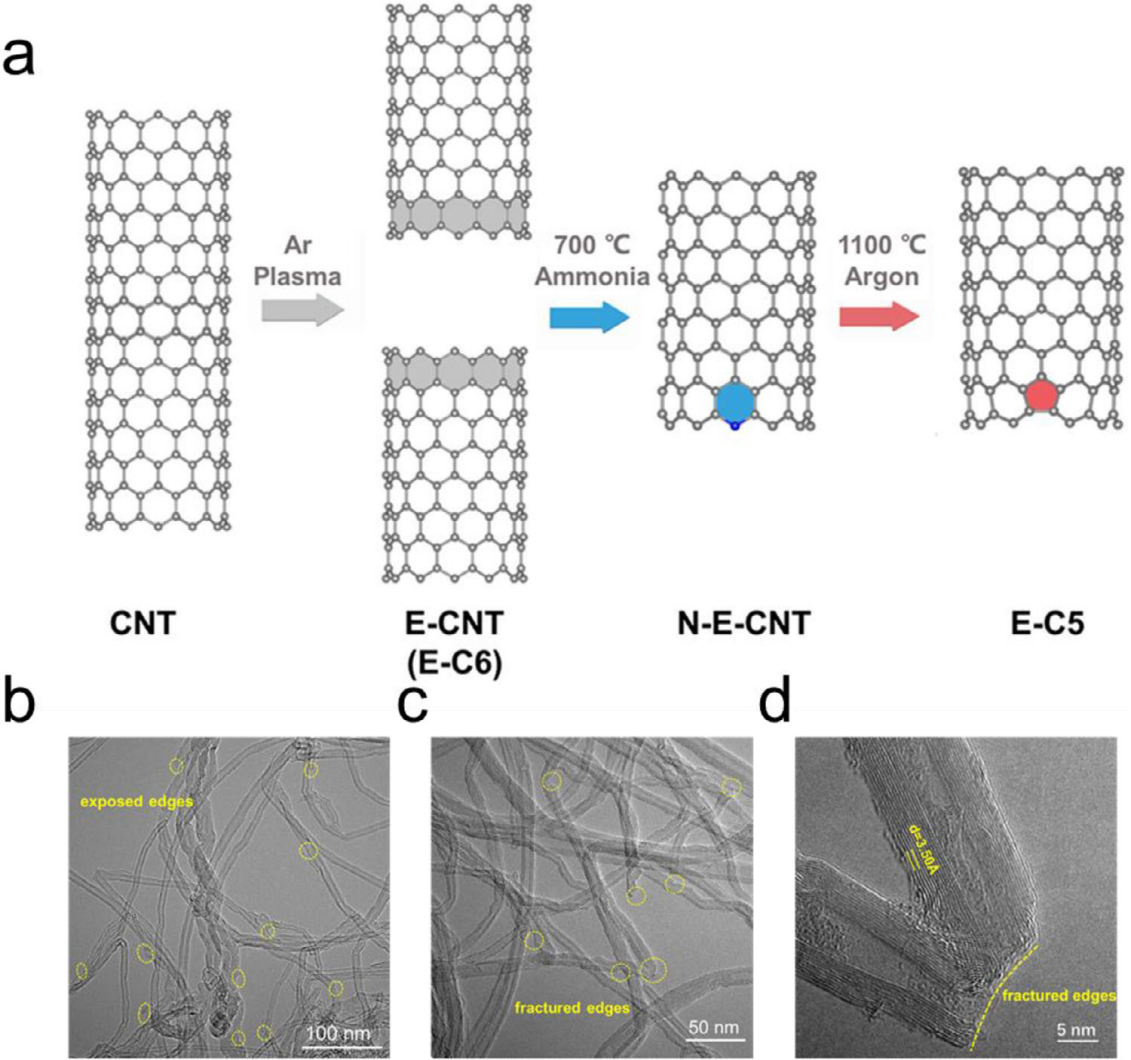
Synthetic illustration and morphology characterizations. a) Schematic illustration of the preparation strategy for E‐C5 and E‐C6. b, c) HRTEM images of E‐C5. d) AC‐HAADF‐STEM image.

To experimentally investigate the effect of pentagon defects on spin polarization and catalytic performance of CMFCs, E‐C5 was designed and prepared through edge reconstruction by specific N dopants subtraction (**Figure** **2**a).^[^ ^36^ ^]^ Generally, the removal of graphitic N would convert to divacancy defects, and pyridinic N or pyrrolic N would convert to pentagon defects.^[^ ^36^, ^37^ ^]^ Thus, in order to introduce pentagon defects on the edge, the edge‐defect‐rich carbon nanotube (E‐CNT or named E‐C6) was first synthesized by argon plasma etching, which is an appropriate method to induce edge defects without the introduction of extra heteroatoms.^[^ ^38^, ^39^ ^]^ After the argon plasma etching, the following E‐C6 was annealed at 700 °C for 3 h in the ammonia flow to lead the nitrogen doping at the edge of the carbon materials.^[^ ^40^ ^]^ Subsequently, the nitrogen‐doped E‐CNT (N‐E‐CNT) was annealed at 1100 °C in an argon atmosphere for 2 h to remove nitrogen species and get pentagon defects. Transmission electron microscopy (TEM) was applied to analyze the morphology characterization of E‐C6 (Figure S5, Supporting Information) and E‐C5. As depicted in Figure 2b,c, an increased number of edges were exposed, and the fractured edges were clearly discernible, reflecting that the pristine CNTs were broken into shorter ones and more edge defects were introduced in E‐C5 (Figure 2d). Additionally, the spherical aberration‐corrected high‐angle annular dark‐field scanning transmission electron microscopy (AC‐HAADF‐STEM) was applied to get more structural information from the atomic level, and the corresponding Fourier transform fitting was performed to verify the existence of pentagon defects. As exhibited in Figure S6 (Supporting Information), the yellow lines represented hexagons and the red lines represented the pentagon defects, and these images confirm the introduction of pentagon defects in E‐C5.

**Figure 3:**
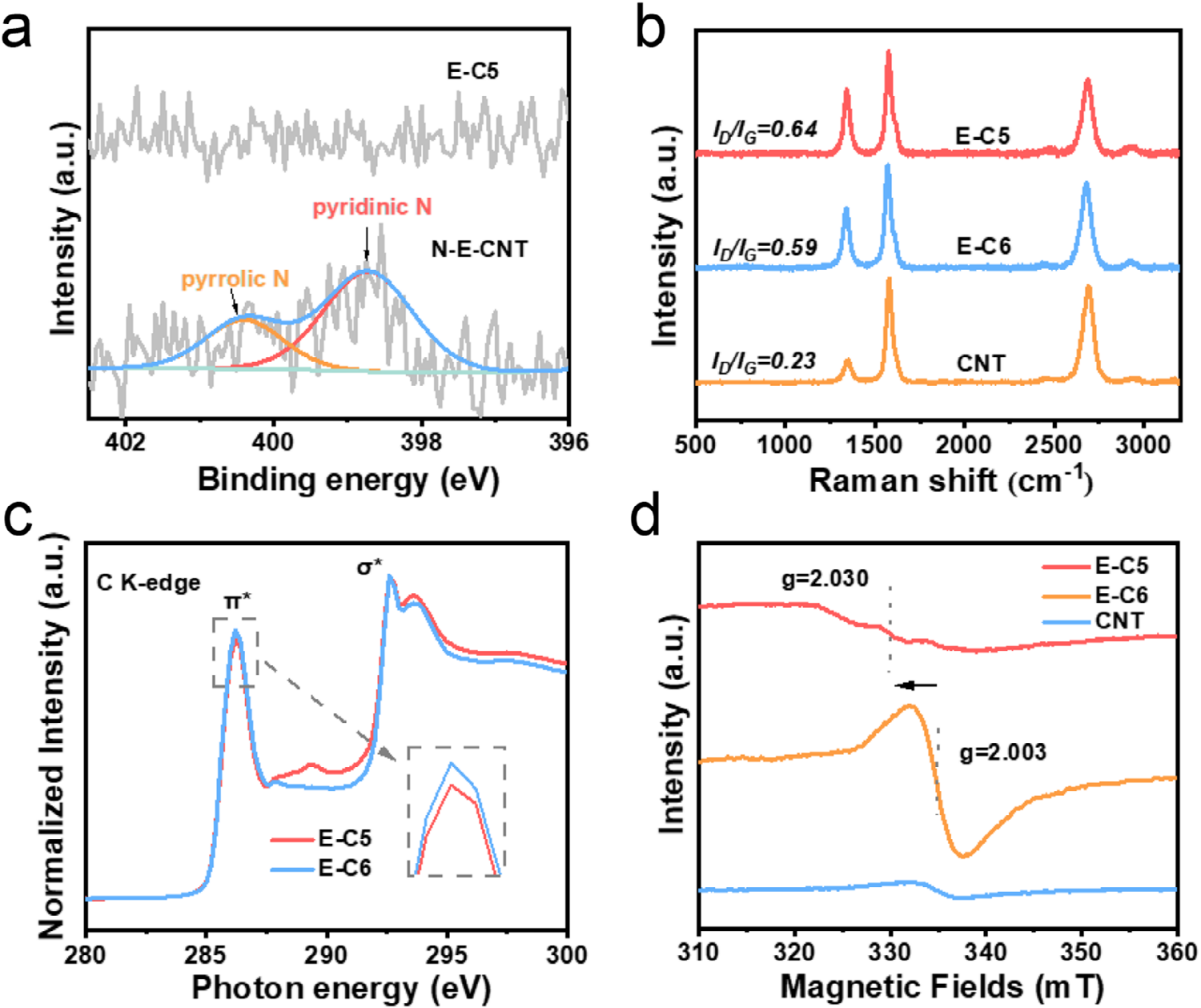
Structure characterizations of CNTs. a) High‐resolution N 1s XPS spectra of N‐E‐CNT and E‐C5. b) Raman spectra of E‐C5, E‐C6, and CNT. c) C K‐edge XANES of E‐C5 and E‐C6. d) EPR spectra of E‐C5, E‐C6, and CNT.

The nitrogen species were subsequently analyzed by using X‐ray photoelectron spectroscopy (XPS) spectra, which indicated that the N‐E‐CNT was dominated by the pyridinic nitrogen (398.5 eV) and pyrrolic nitrogen (400.5 eV), without graphitic N (401 eV) (**Figure** **3**a). The N 1s XPS spectra of E‐C5 showed no signals in the range of 389–403 eV, indicating the nitrogen dopants were removed from N‐E‐CNT. The elimination of pyridinic N and pyrrolic N demonstrated the successful introduction of pentagon defects.^[^ ^32^ ^]^ As shown in Figure S8 (Supporting Information), the X‐ray diffraction (XRD) displayed typical defective carbon features with two broad peaks of (002) and (101), and the peak intensity of E‐C5 was reduced, suggesting a lower graphitization and a higher concentration of defects in E‐C5 compared to CNT and E‐C6.^[^ ^41^ ^]^ The carbon structural features were investigated by Raman spectra, where the ratios between defect carbon (D band, 1350 cm^−1^) and graphitic sp^2^ carbon (G band, 1580 cm^−1^) represented the defect density.^[^ ^42^, ^43^ ^]^ The *I_D_/I_G_* ratio showed an increased level (E‐C6 < E‐C5), implying more defects were generated after the removal of pyridinic nitrogen and pyrrolic nitrogen (Figure 3b). Moreover, a noticeable D’ peak was detected on the Raman spectra of E‐C5 and E‐C6 (Figure S8, Supporting Information), confirming the generation of edge defects after argon plasma etching.^[^ ^44^ ^]^. The defective nature of E‐C5 was further affirmed by the corresponding increasing specific surface area (Figure S9, Supporting Information). In the near‐edge X‐ray absorption fine structure (NEXAFS) spectra (Figure 3c), the sharp peaks at around 285.5 and 293.8 eV are attributed to C 1s → π^*^ and σ^*^ transitions, respectively.^[^ ^27^, ^36^ ^]^ The intensity of the π^*^ state in E‐C5 was observed to be weaker than that in E‐C6, suggesting that the introduction of pentagon defects through edge reconstruction damaged the sp^2^‐hybridized carbon structure and disturbed the overall π^*^ conjugation during the N removal process.^[^ ^36^, ^37^ ^]^ Of particular interest, electron paramagnetic resonance (EPR) is an effective measurement to provide insights into the spin characteristics of carbon materials, since it can detect unpaired spins and conduction electrons.^[^ ^45^ ^]^ As shown in Figure 3d, the peak intensity of E‐C6 was stronger compared to CNT, reflecting the edge defects in the carbon materials introduced a high density of unpaired electrons, rendering them susceptible to external magnetic fields.^[^ ^27^, ^46^ ^]^ However, the g‐value of E‐C5 was significantly increased, and the peak signal of E‐C5 changed into irregular resonance after the pentagon defects located at the edge, which could be ascribed to the spin dipole−dipole interaction.^[^ ^47^ ^]^ These results illustrate that the edge reconstruction by specific N dopants subtraction can introduce pentagon defects, which could induce spin polarization at the edge of CMFCs.

### 2.2 Catalytic Performance in Fenton‐Like Reaction

**Figure 4:**
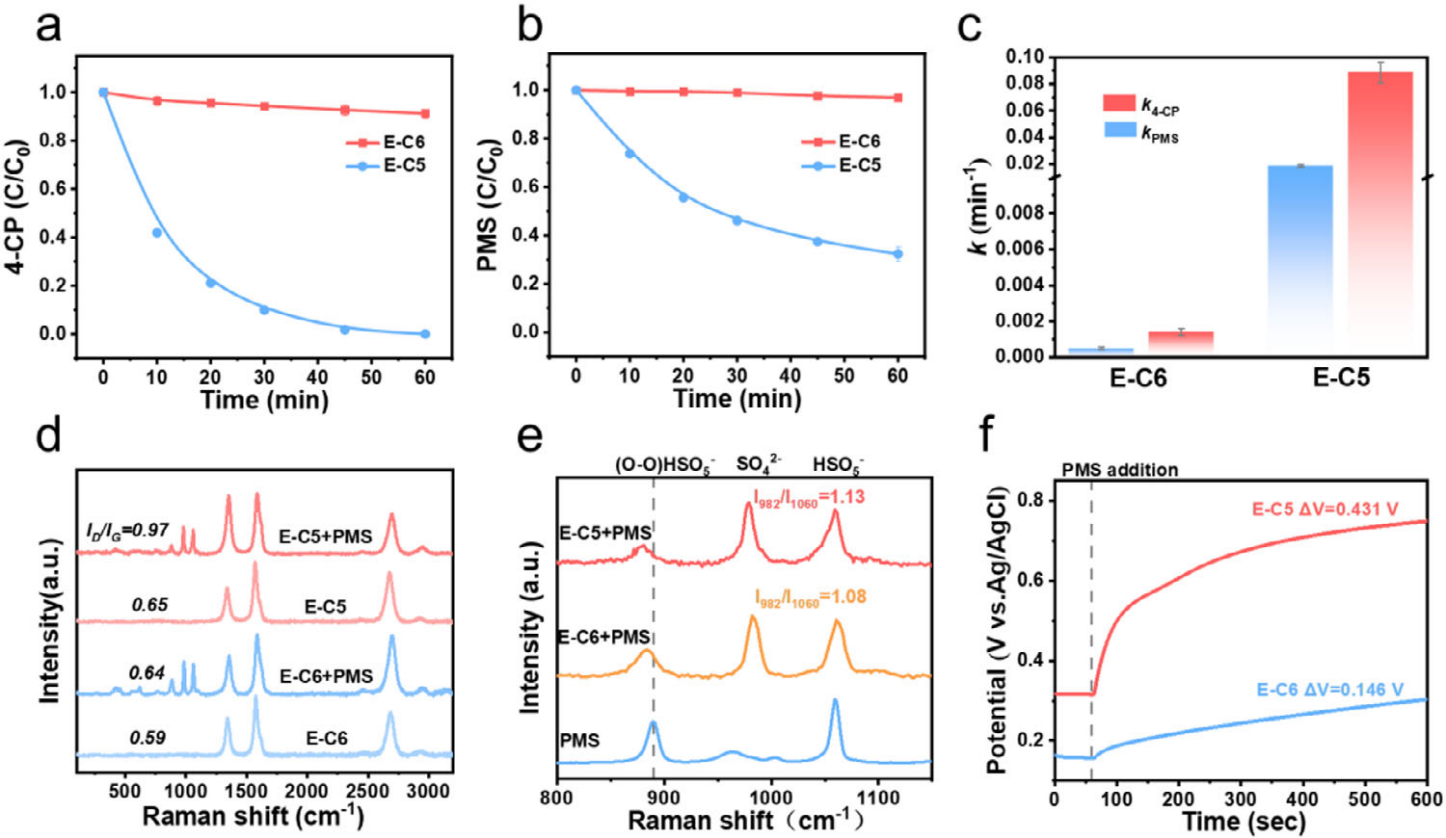
Catalytic performance of the E‐C5/PMS and E‐C6/PMS systems. a) Catalytic activities of E‐C5 and E‐C6 by PMS activation for 4‐CP removal. b) PMS consumption capability of E‐C5 and E‐C6. c) Comparison of 4‐CP degradation rate and PMS consumption rate by E‐C5 and E‐C6/PMS systems. d, e) In situ Raman spectra for E‐C5 and E‐C6 combined with PMS for 30 s. f) The changes of open‐circuit potentials of E‐C5‐PMS and E‐C6‐PMS complexes. Reaction condition: [4‐CP] = 10 mg L^−1^, [PMS] = 1.5 mm, [catalyst] = 0.1 g L^−1^.

To elucidate the structure‐activity relationship between spin polarization and catalytic performance, catalytic activities of the E‐C5 and E‐C6 were evaluated in activating PMS for oxidative degradation of 4‐chlorophenol (4‐CP). Although the adsorption efficiency of E‐C5 and E‐C6 for 4‐CP was equally modest, yielding a removal ratio of less than 20% (Figure S11, Supporting Information), E‐C6 could hardly activate PMS to remove 4‐CP (**Figure** **4**a). Surprisingly, after the spin polarization, the activities of the E‐C5 boosted a lot. The rapid 4‐CP removal on E‐C5/PMS within 60 min was successfully realized (Figure 4a), which showed higher Fenton‐like activity than the pristine CNT (Figure S12, Supporting Information). To exclude the influence of the annealing process on the catalyst, E‐C6 was further annealed at 1100 °C for 2 h to fabricate E‐C6‐1100. Only 10% 4‐CP was degraded by the E‐C6‐1100/PMS system (Figure S13, Supporting Information), indicating the poor PMS activation performance by E‐C6‐1100. These distinct phenomena reflected that the existence of pentagon defects was the dominant factor in accelerating PMS activation. In order to get a clear comparison of the activities, the 4‐CP degradation kinetics were fitted by the pseudo‐first‐order model (Figure S14, Supporting Information). It was noteworthy that the *k*_4‐CP_ of E‐C5 (0.0886min^−1^) was as high as 63‐fold of E‐C6 (0.0014min^−1^). To deeply compare the difference between E‐C5 and E‐C6 in activating PMS, the decomposition of PMS was measured. As shown in Figure 4b, the decomposition of PMS in the E‐C5/PMS system was greater than the other, with a 70% PMS decomposition ratio in 60 min. The enhanced degradation of 4‐CP and the improved activation of PMS were both observed in E‐C5 compared to E‐C6 (Figure 4c).

Furthermore, in situ Raman spectra were adopted to assess the ability of PMS consumption. As shown in Figure 4d, the *I*_D_/*I*_G_ ratios of E‐C5 and E‐C6 increased from 0.64 and 0.59 to 0.97 and 0.65, respectively, after the addition of PMS. The adsorption of PMS in carbocatalysts would interrupt the homogeneous π system of sp^2^ carbons and induce local electron exhaustion.^[^ ^18^ ^]^ Therefore, E‐C5 achieved a greater increased value of *I*_D_/*I*_G_ (0.33), reflecting a superior adsorption quantity for PMS than that of E‐C6 (0.05). Notably, the HSO_5_^−^ peak at 889 cm^−1^ showed an obvious blueshift of 5 cm^−1^ after adding PMS to the E‐C5 and E‐C6 in situ Raman spectra (Figure 4e). The blueshift of HSO_5_^−^ could be ascribed to the prolonged peroxo O─O bond for the PMS^*^ intermediate in E‐C5/PMS and E‐C6/PMS systems.^[^ ^48^, ^49^ ^]^ Additionally, the value of I_982_/I_1060_ could be regarded as an indicator for the consumption of PMS, and the higher value suggests the greater consumption of PMS.^[^ ^50^, ^51^ ^]^ Compared to E‐C6, E‐C5 exhibited an increase in the I_982_/I_1060_ value from 1.08 to 1.13, indicating that spin polarization promoted the optimal adsorption of PMS and accelerated the decomposition of HSO_5_^−^ to SO_4_^2−^. Moreover, the dynamic Raman spectra proved the extremely fast decomposition of PMS in 90 s (Figure S16, Supporting Information), reflecting that E‐C5 showed the remarkable capability to accelerate the decomposition of PMS to produce more reactive oxygen species. As exhibited in Figure 4f, the in situ measurements of open‐circuit potential were taken to estimate the PMS adsorption ability. The higher potential increase was observed in E‐C5/PMS (0.431 V) compared to E‐C6/PMS (0.146 V), suggesting a stronger PMS adsorption ability. Therefore, combining the results of 4‐CP degradation with PMS activation, it is apparent that E‐C5, with spin polarization, exhibits enhanced performance in the decomposition of PMS and removal of 4‐CP.

### 2.3 Mechanism Investigation of the E‐C5/PMS System

**Figure 5:**
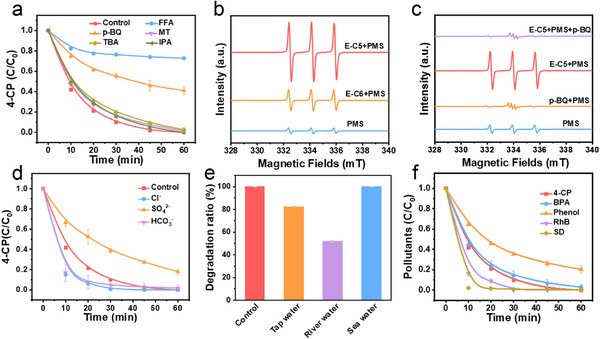
Mechanism investigation of E‐C5/PMS systems. a) The degradation curves of 4‐CP by the E‐C5/PMS system in the presence of diverse scavengers. b, c) EPR spectra obtained by spin trapping with TEMP (water as the solvent). d) The degradation curves of 4‐CP in the presence of various inorganic anions. e) The degradation ratio of 4‐CP removal in the real water systems. f) Degradation curves of different pollutants in the E‐C5/PMS system. Reaction condition: [Cl^−^] = [SO_4_^2−^] = [HCO_3_^−^] = 10 mm, [4‐CP] = [BPA] = [Phenol] = [RhB] = [SD] = 10 mg L^−1^, [FFA] = [p‐BQ] = 7.5 mm, [MT] = 750 mm, [TBA] = [IPA] = 500 mm, [PMS] = 1.5 mm, [catalyst] = 0.1 g L^−1^.

In order to figure out what the real reactive species are and gain an in‐depth understanding of the activation mechanism in the E‐C5/PMS systems, various experiments were employed. In the quenching test, methanol (MT) was applied as the quenching agent of hydroxyl (·OH) and sulfate radicals (SO_4_·^−^), TBA (tertiary butanol) and IPA (isopropanol) were regarded as a selective quenching agent for ·OH, furfuryl alcohol (FFA) was selected as the singlet oxygen (^1^O_2_) quencher, and p‐benzoquinone (p‐BQ) was utilized to capture O_2_^·−^.^[^ ^52^, ^53^ ^]^ As illustrated in **Figure** **5**a, there was a slight influence after adding MT, TBA, and IPA, excluding the role of the ·OH and SO_4_·^−^radicals in PMS activation over E‐C5 for 4‐CP degradation. However, when FFA and p‐BQ were applied, the 4‐CP removal efficiency significantly decreased, and the 4‐CP degradation rate dropped from 0.886 to 0.044 min^−1^ and 0.0143 min^−1^, respectively (Figure S17, Supporting Information). Significantly, when the concentration of p‐BQ reached 15 mm (Figure S18, Supporting Information), the E‐C5/PMS system became inactive (inhibition rate: 91.8%), suggesting that the superoxide radical makes a great contribution to the pollutant oxidation. Thus, the high degradation ratio of 4‐CP did not rely on the ·OH and SO_4_·^−^, but the ^1^O_2_ and O_2_^·−^ played an important role in the E‐C5/PMS system. Subsequently, EPR was measured to further investigate the active species, and 5,5‐dimethyl‐1‐pyrroline (DMPO) and tetramethylpiperidine (TEMP) were individually used as the spin traps for radicals and ^1^O_2_. As shown in Figure S19 (Supporting Information), there were no signals after the addition of DMPO. Nevertheless, there was a characteristic signal of TEMP‐^1^O_2_ with triple peaks when TEMP was added (Figure 4b). Interestingly, the TEMP‐^1^O_2_ signal intensity in the E‐C5/PMS system was much stronger than that in the E‐C6/PMS system, suggesting the efficient ^1^O_2_ generation of the E‐C5/PMS system. The production of ^1^O_2_ in the E‐C5/PMS system was further supported by 1,3‐diphenylisobenzofuran (DPBF) degradation (Figure S20, Supporting Information).^[^ ^54^ ^]^ The peak of DPBF at 411 nm was quickly decreased after the reaction between PMS and E‐C5, reflecting the higher production of ^1^O_2_ in the E‐C5/PMS system. Since the existence of ^1^O_2_ in the E‐C5/PMS system and the addition of p‐BQ could suppress the degradation of 4‐CP, it was crucial to determine whether there was O_2_^·−^ in the system. The signals of DMPO‐O_2_^·−^ were detected in methanol solution with sext peaks (Figure S21, Supporting Information), which implied the existence of O_2_^·−^ in the E‐C5/PMS system. To deeply verify the relationship between ^1^O_2_ and O_2_^·−^, p‐BQ was added in the E‐C5/TEMP system, and the signal of TEMP‐^1^O_2_ with triple peaks disappeared (Figure 5c), revealing the reactive species ^1^O_2_ generated from O_2_^·−^. On the basis of the above analyses, a ^1^O_2‐_dominated nonradical catalytic mechanism for the oxidative degradation of 4‐CP in the E‐C5/PMS system was affirmed.

Since ^1^O_2_ shows strong resistance against environmental interference,^[^ ^55^ ^]^ the applications of water decontamination in the E‐C5/PMS system were investigated. As shown in Figure S22 (Supporting Information), only a slight decrease was observed when the pH value was adjusted from 4 to 9, reflecting that the E‐C5/PMS system possessed a considerable pH resilience. Furthermore, the interference of inorganic anions was tested (Figure 5d), and more than 80% 4‐CP was removed in the E‐C5/PMS system in the solution of various inorganic anions (SO_4_^2−^, HCO_3_^−^ and Cl^−^), which is superior to the homogenous singlet oxygenation process (ClO^−^ /PMS system) (Figure S23, Supporting Information). Even in the real water systems (like tap water, lake water, and sea water), the E‐C5/PMS system could still maintain high degradation performance of 4‐CP (Figure 5e), reflecting the great resistance to interference of the E‐C5/PMS system. Meanwhile, the E‐C5/PMS system exhibited outstanding removal performance for other pollutants (Figure 5f), including phenol, rhodamine B (RhB), bisphenol A (BPA), and sulfadiazine (SD). Additionally, the stability of E‐C5 was investigated. Although the 4‐CP degradation performance was reduced after the cycle, the activity could be recovered by regenerating in an Ar atmosphere at 700 °C for 1 h (Figure S24, Supporting Information).

### 2.4 Theoretical Calculations for PMS Activation

**Figure 6:**
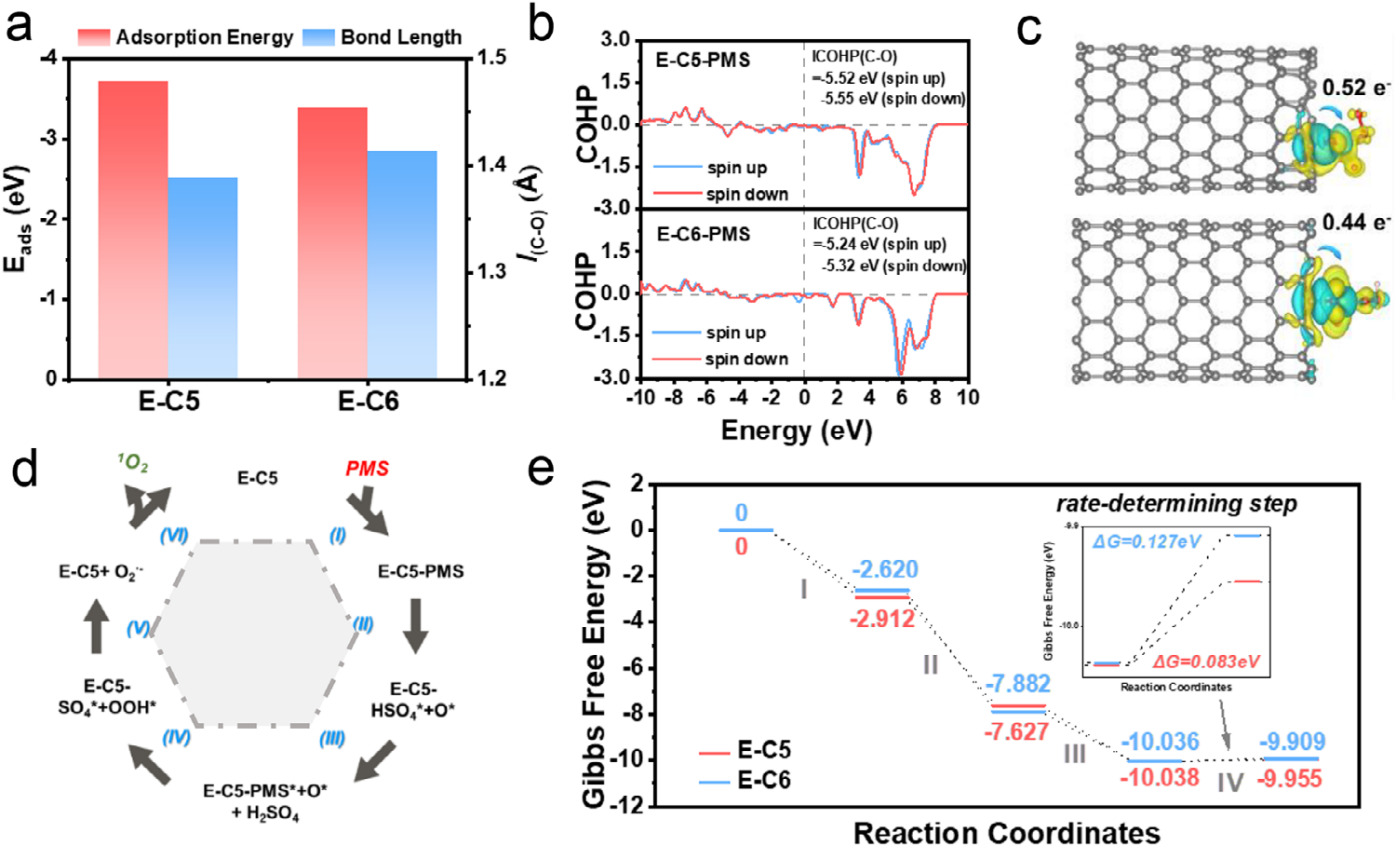
Revealing the effect of spin polarization on the Fenton‐like activity. a) Adsorption energy of PMS on the C sites of E‐C6 and E‐C5 and the lengths of the C─O bond. b) The COHP of E‐C5 and E‐C6 after absorbing PMS. c) Differential charge of E‐C6 and E‐C5 after adsorption of the PMS molecule, and the corresponding charge transfer. The cyan and yellow regions represent areas of electron deficiency and areas of increased electron density, respectively. d) Proposed reaction process for PMS activation to ^1^O_2_ on E‐C5 catalysts. e) The calculated potential energy diagrams for the reaction coordinates on E‐C5 and E‐C6.

DFT calculations were performed to further grasp the contribution of spin polarization in E‐C5 for PMS activation. The probable adsorption sites of E‐C5 and E‐C6 for PMS were shown in Figure S25 (Supporting Information). As expected, the E‐C5 showed higher adsorption energy (E_ads_) (−3.72 eV) compared with E‐C6 (−3.39 eV), revealing the stronger interaction between PMS and E‐C5 (**Figure** **6**a). Moreover, it was noticed that the C─O bond between the PMS molecule and E‐C5 (1.389 Å) was shorter than E‐C6 (1.413 Å), consisting with the higher interaction between the PMS molecule and E‐C5. Furthermore, the crystal orbital Hamilton population (COHP) of E‐C5 and E‐C6 was established to explore the contribution of spin polarization (Figure 6b), and it showed a higher absolute value of calculated integrated COHP for E‐C5/PMS (−5.55 eV) than E‐C6/PMS (−5.32 eV). Notably, spin‐down electrons contributed more to the formation of the C─O bond compared to the spin‐up state electrons, revealing that spin‐down electrons were more prone to strengthen the bond and improve the adsorption of PMS. Additionally, a significant electron transfer was observed between PMS and E‐C5 as displayed from the charge density and Bader charge analysis (Figure 6c), suggesting the adsorbed site in E‐C5 was prone to transfer electrons to PMS. The Bader charge decreased from 4.12e to 3.60e after adsorption of PMS on the active site, and the direction of electron transfer was proved by the in situ Raman spectra (Figure S26, Supporting Information).

Combining with the nonradical mechanism and the analysis of the relationship between E‐C5 and PMS, the probable reaction pathway to product ^1^O_2_ was elucidated by the DFT calculation. The preferred reaction configurations and potential reaction paths are shown in Figure S27 (Supporting Information) and Figure 6d, respectively, namely, PMS→ PMS^*^→ HSO_4_^*^+O^*^→ PMS^*^+O^*^→ SO_4_^*^+OOH^*^→ O_2_^·−^→^1^O_2_. First, PMS was adsorbed on the E‐C5, and divided into HSO_4_^*^ and O^*^, which could be verified by the elongated O─O bond. Meanwhile, HSO_4_^*^ was dissociated to form H_2_SO_4_ via an exothermic reaction.^[^ ^56^ ^]^ This reaction pathway was confirmed by the fact that higher concentrations of SO_4_^2−^ inhibited the activation of PMS (Figure 5d). Additionally, according to the in situ Raman spectra, after the adsorption of PMS for 90 s, the peak of HSO_5_^−^ was absent, and the peak of SO_4_^2−^ was higher than before. It was not only an indicator for the consumption of PMS, but also confirmed the existence of SO_4_^2−^ during the reaction. Subsequently, another PMS was adsorbed on the E‐C5 to form OOH^*^ and SO_4_^*^. Following this, OOH^*^ was utilized to generate O_2_^·−^.^[^ ^57^ ^]^ Given the improved PMS activation performance of E‐C5 under acid conditions, it could be inferred that ^1^O_2_ was produced by two O_2_^·−^ (2O_2_^·−^ + 2H^+^ = ^1^O_2_ + H_2_O_2_). Based on the proposed pathways for ^1^O_2_ generation, the Gibbs free energy evolutions of each step involved in the activation of PMS on E‐C5 and E‐C6 were computed (Figure 6e). It could be noticed that both the free energy diagrams of E‐C5 and E‐C6 showed the largest energy barrier for the OOH^*^ producing process, suggesting that the formation of the OOH^*^ intermediate was the rate‐determining step. The energy barrier of this rate‐determining step was reduced from 0.127 eV (E‐C6) to 0.083 eV (E‐C5), demonstrating E‐C5 was more favorable to generate ^1^O_2_. The aforementioned results uncovered that the spin polarization induced by the introduction of a pentagon defect at the edge would facilitate electron transfer between the PMS molecular and the carbon catalyst, and thermodynamically promote the formation of the ^*^OOH intermediate, which is conducive to efficient ^1^O_2_ production.

## 3 Conclusion

In summary, we have proposed a novel spin polarization strategy through the introduction of topological pentagon defects into carbon‐based metal‐free catalysts. Specifically, the E‐C5 catalyst, characterized by edged pentagon defects, exhibits a greater number of spin‐down electrons located below the Fermi level. The DFT calculations elucidated that, benefiting from the spin polarization, E‐C5 showed higher adsorption ability and efficient electron transfer with PMS. Furthermore, the energy of the rate‐determining step of the key OOH^*^ intermediate was reduced, endowing E‐C5 with unique proficiency in generating ^1^O_2_. Consequently, this approach effectively accelerates the PMS activation and achieves a higher Fenton‐like activity in the E‐C5/PMS system, which is 63‐fold of that of the E‐C6/PMS system. These findings not only offer a controllable strategy for spin polarization in carbon catalysis but also deepen our understanding of the application of spin polarization in Fenton‐like reactions. Significantly, it is the first time to manipulate spin polarization to boost activation of carbon catalysts by topological pentagon defect engineering, which provided a brand‐new insight into the rational invention of highly efficient carbon catalysts.

## 4 Experimental Section

### Chemicals and Materials

The following reagents were used as received: p‐chlorophenol (4‐CP), sulfamethoxazole (SMX), rhodamine B (RhB), bisphenol A (BPA), phenol, furfuryl alcohol (FFA), (1,4‐Benzoquinone) p‐BQ, 2,2′‐azino‐bis (3‐ethylbenzothiazoline‐6‐sulfonic acid) diammonium salt (ABTS), potassium iodide (KI), 2,2,6,6‐tetramethyl‐4‐piperidinol (TEMP, ≥98%), methanol, ethanol, and acetone, isopropanol (IPA), tert‐Butanol (TBA) were purchased from Aladdin. 5,5‐dimethyl‐1‐pyrrolidine N‐oxide (DMPO, 98%) was obtained from Dojindo. Potassium peroxymonosulfate (PMS, KHSO_5_) was obtained from Damas‐beta. Phosphate buffer solutions (saline‐free) were obtained from Yuanye. Nafion solution (5 wt.%) was purchased from Sigma–Aldrich. Ultrapure deionized water (>18 MΩ·cm), produced with a Millipore system, was used for the preparation of all experimental solutions. All chemicals were of reagent grade and were used without further purification or treatment.

### Synthesis of E‐CNT

E‐CNT (E‐C6) was fabricated from a G‐MWCNT by plasma treatment (NE‐PE02, Shenzhen Naen Tech Co., Ltd, China) in 60 min under an atmosphere of argon at 350 W.

### Synthesis of N‐E‐CNT

The E‐CNT was annealed at 700 °C for 3 h in the ammonia flow to give N‐E‐CNT. This was further annealed at 1100 °C for 2 h under argon to obtain E‐C5.

In all experiments performed, no unexpected or unusually high safety hazards were encountered.

## Conflict of Interest

The authors declare no conflict of interest.

## Supporting information



Supporting Information

## Data Availability

The data that support the findings of this study are available from the corresponding author upon reasonable request.

## References

[1] G. Gurung , M. Elekhtiar , Q.‐Q. Luo , D.‐F. Shao , E. Y. Tsymbal , Nat. Commun. 2024, 15, 10242. 39592583 10.1038/s41467-024-54526-1 PMC11599937

[2] L. Li , J. Zhou , X. Wang , J. Gracia , M. Valvidares , J. Ke , M. Fang , C. Shen , J. M. Chen , Y. C. Chang , C. W. Pao , S. Y. Hsu , J. F. Lee , A. Ruotolo , Y. Chin , Z. Hu , X. Huang , Q. Shao , Adv. Mater. 2023, 35, 2302966. 10.1002/adma.202302966 37436805

[3] Y.‐Z. Jin , Z. Li , J.‐Q. Wang , R. Li , Z.‐Q. Li , H. Liu , J. Mao , C.‐K. Dong , J. Yang , S.‐Z. Qiao , X.‐W. Du , Adv. Energy Mater. 2018, 8, 1703469.

[4] R. R. Chen , G. Chen , X. Ren , J. Ge , S. J. H. Ong , S. Xi , X. Wang , Z. J. Xu , Angew. Chem., Int. Ed. 2021, 60, 25884. 10.1002/anie.202109065 34561927

[5] Z. Du , Z. Meng , X. Gong , Z. Hao , X. Li , H. Sun , X. Hu , S. Yu , H. Tian , Angew. Chem., Int. Ed. 2024, 136, 202317022. 10.1002/anie.202317022 38151463

[6] X. Ren , T. Wu , Y. Sun , Y. Li , G. Xian , X. Liu , C. Shen , J. Gracia , H. J. Gao , H. Yang , Z. J. Xu , Nat. Commun. 2021, 12, 2608. 33972558 10.1038/s41467-021-22865-y PMC8110536

[7] Y. Dai , B. Liu , Z. Zhang , P. Guo , C. Liu , Y. Zhang , L. Zhao , Z. Wang , Adv. Mater. 2023, 35, 2210757. 10.1002/adma.202210757 36773335

[8] Y. Zhao , H. Wang , J. Li , Y. Fang , Y. Kang , T. Zhao , C. Zhao , Adv. Funct. Mater. 2023, 33, 2305268.

[9] C.‐C. Lin , T.‐R. Liu , S.‐R. Lin , K. M. Boopathi , C.‐H. Chiang , W.‐Y. Tzeng , W. C. Chien , H.‐S. Hsu , C.‐W. Luo , H.‐Y. Tsai , H.‐A. Chen , P.‐C. Kuo , J. Shiue , J.‐W. Chiou , W.‐F. Pong , C.‐C. Chen , C.‐W. Chen , J. Am. Chem. Soc. 2022, 144, 15718. 35975916 10.1021/jacs.2c06060

[10] Y. Bao , J. Xiao , Y. Huang , Y. Li , S. Yao , M. Qiu , X. Yang , L. Lei , Z. Li , Y. Hou , G. Wu , B. Yang , Angew. Chem., Int. Ed. 2024, 63, 202406030. 10.1002/anie.202406030 39020457

[11] C.‐H. Chiang , C.‐C. Lin , Y.‐C. Lin , C.‐Y. Huang , C.‐H. Lin , Y.‐J. Chen , T.‐R. Ko , H.‐L. Wu , W.‐Y. Tzeng , S.‐Z. Ho , Y.‐C. Chen , C.‐H. Ho , C.‐J. Yang , Z.‐W. Cyue , C.‐L. Dong , C.‐W. Luo , C.‐C. Chen , C.‐W. Chen , J. Am. Chem. Soc. 2024, 146, 23278. 39049154 10.1021/jacs.4c05798 PMC11345765

[12] D. Zhang , Y. Li , P. Wang , J. Qu , S. Zhan , Y. Li , Angew. Chem., Int. Ed. 2023, 62, 202303807. 10.1002/anie.202303807 37062701

[13] Z. Zhang , P. Ma , L. Luo , X. Ding , S. Zhou , J. Zeng , Angew. Chem., Int. Ed. 2023, 62, 202216837. 10.1002/anie.202216837 36598399

[14] X. Liu , L. Dai , Nat. Rev. Mater. 2016, 1, 16064.

[15] X. Yan , Y. Jia , X. Yao , Chem. Soc. Rev. 2018, 47, 7628. 30246207 10.1039/c7cs00690j

[16] Y. Jia , X. Yao , Acc. Chem. Res. 2023, 56, 948. 36989384 10.1021/acs.accounts.2c00809

[17] J. Ortiz‐Medina , Z. Wang , R. Cruz‐Silva , A. Morelos‐Gomez , F. Wang , X. Yao , M. Terrones , M. Endo , Adv. Mater. 2019, 31, 1805717. 10.1002/adma.201805717 30687977

[18] W. Ren , G. Nie , P. Zhou , H. Zhang , X. Duan , S. Wang , Environ. Sci. Technol. 2020, 54, 6438. 32302479 10.1021/acs.est.0c01161

[19] Y. Hu , D. Chen , S. Wang , R. Zhang , Y. Wang , M. Liu , Sep. Purif. Technol. 2022, 280, 119791.

[20] Y. Wang , Z. Zhang , Z. Yin , Z. Liu , Y. Liu , Z. Yang , W. Yang , Appl. Catal. B Environ. 2022, 319, 121891.

[21] G. Ye , S. Liu , K. Huang , S. Wang , K. Zhao , W. Zhu , Y. Su , J. Wang , Z. He , Adv. Funct. Mater. 2022, 32, 2111396.

[22] I. Y. Jeon , S. Zhang , L. Zhang , H. J. Choi , J. M. Seo , Z. Xia , L. Dai , J. B. Baek , Adv. Mater. 2013, 25, 6138. 24038522 10.1002/adma.201302753

[23] J. Liang , Y. Jiao , M. Jaroniec , S. Z. Qiao , Angew. Chem., Int. Ed. 2012, 51, 11496. 10.1002/anie.201206720 23055257

[24] E. Wimmer , A. J. Freeman , in Handbook of Surface Science, (Eds: K. Horn , M. Scheffler ), 2, Elsevier, Amsterdam, The Netherland 2000, pp. 1–91.

[25] H. Wang , T. Maiyalagan , X. Wang , ACS Catal. 2012, 2, 781.

[26] S. K. Singh , K. Takeyasu , J. Nakamura , Adv. Mater. 2019, 31, 1804297. 10.1002/adma.201804297 30350433

[27] C. Zhang , W. Shen , K. Guo , M. Xiong , J. Zhang , X. Lu , J. Am. Chem. Soc. 2023, 145, 11589. 37158560 10.1021/jacs.3c00689

[28] J. Zhu , Y. Huang , W. Mei , C. Zhao , C. Zhang , J. Zhang , I. S. Amiinu , S. Mu , Angew. Chem., Int. Ed. 2019, 58, 3859. 10.1002/anie.201813805 30637898

[29] Y. Dong , Q. Zhang , Z. Tian , B. Li , W. Yan , S. Wang , K. Jiang , J. Su , C. W. Oloman , E. L. Gyenge , R. Ge , Z. Lu , X. Ji , L. Chen , Adv. Mater. 2020, 32, 2001300. 10.1002/adma.202001300 32490580

[30] Q. Zhou , C. Song , P. Wang , Z. Zhao , Y. Li , S. Zhan , Proc. Natl. Acad. Sci. USA 2023, 120, 2300085120. 10.1073/pnas.2300085120 PMC10068799 36952382

[31] W. Ren , C. Cheng , P. Shao , X. Luo , H. Zhang , S. Wang , X. Duan , Environ. Sci. Technol. 2022, 56, 78. 34932343 10.1021/acs.est.1c05374

[32] H. Xia , R. Pang , X. Dong , Q. Liu , J. Chen , E. Wang , J. Li , J. Am. Chem. Soc. 2023, 145, 25695. 37943722 10.1021/jacs.3c08556

[33] G. Chen , M. Isegawa , T. Koide , Y. Yoshida , K. Harano , k. Hayashida , S. Fujita , K. Takeyasu , K. Ariga , J. Nakamura , Angew. Chem., Int. Ed. 2024, 63, 202410747. 10.1002/anie.202410747 39305103

[34] G. Z. Magda , X. Jin , I. Hagymasi , P. Vancso , Z. Osvath , P. Nemes‐Incze , C. Hwang , L. P. Biro , L. Tapaszto , Nature 2014, 514, 608. 25355361 10.1038/nature13831

[35] J. Yu , H. Feng , L. Tang , Y. Pang , G. Zeng , Y. Lu , H. Dong , J. Wang , Y. Liu , C. Feng , J. Wang , B. Peng , S. Ye , Prog. Mater Sci. 2020, 111, 100654.

[36] X. Wang , Y. Jia , X. Mao , L. Zhang , D. Liu , L. Song , X. Yan , J. Chen , D. Yang , J. Zhou , K. Wang , A. Du , X. Yao , Chem 2020, 6, 2009.

[37] Y. Jia , L. Zhang , L. Zhuang , H. Liu , X. Yan , X. Wang , J. Liu , J. Wang , Y. Zheng , Z. Xiao , E. Taran , J. Chen , D. Yang , Z. Zhu , S. Wang , L. Dai , X. Yao , Nat. Catal. 2019, 2, 688.

[38] J. H. Zhong , J. Zhang , X. Jin , J. Y. Liu , Q. Li , M. H. Li , W. Cai , D. Y. Wu , D. Zhan , B. Ren , J. Am. Chem. Soc. 2014, 136, 16609. 25350471 10.1021/ja508965w

[39] X. Sun , J. Bao , K. Li , M. D. Argyle , G. Tan , H. Adidharma , K. Zhang , M. Fan , P. Ning , Adv. Funct. Mater. 2020, 31, 2006287.

[40] D. Guo , R. Shibuya , C. Akiba , S. Saji , T. Kondo , J. Nakamura , Science 2016, 351, 361. 26798009 10.1126/science.aad0832

[41] S. Liu , S. Yin , Z. Zhang , L. Feng , Y. Liu , L. Zhang , J. Hazard. Mater. 2023, 441, 129905. 36113348 10.1016/j.jhazmat.2022.129905

[42] J. Wang , J. Yu , Q. Fu , H. Yang , Q. Tong , Z. Hao , G. Ouyang , ACS Cent. Sci. 2021, 7, 355. 33655073 10.1021/acscentsci.0c01600 PMC7908038

[43] Y. Jiang , L. Yang , T. Sun , J. Zhao , Z. Lyu , O. Zhuo , X. Wang , Q. Wu , J. Ma , Z. Hu , ACS Catal. 2015, 5, 6707.

[44] H. Zhong , Z. Gong , J. Yu , Y. Hou , Y. Tao , Q. Fu , H. Yang , X. Xiao , X. Cao , J. Wang , G. Ouyang , Adv. Sci. 2024, 11, 2404958. 10.1002/advs.202404958 PMC11538648 39258821

[45] M. Zaka , Y. Ito , H. Wang , W. Yan , A. Robertson , Y. A. Wu , M. H. Rümmeli , D. Staunton , T. Hashimoto , J. J. L. Morton , A. Ardavan , G. A. D. Briggs , J. H. Warner , ACS Nano 2010, 4, 7708. 21082779 10.1021/nn102602a

[46] Z. Tan , K. Ni , G. Chen , W. Zeng , Z. Tao , M. Ikram , Q. Zhang , H. Wang , L. Sun , X. Zhu , X. Wu , H. Ji , R. S. Ruoff , Y. Zhu , Adv. Mater. 2017, 29, 1603414. 10.1002/adma.201603414 27991689

[47] K. Judai , S. Numao , A. Furuya , J. Nishijo , N. Nishi , J. Am. Chem. Soc. 2008, 130, 1142. 18183984 10.1021/ja078287o

[48] W. Ren , L. Xiong , X. Yuan , Z. Yu , H. Zhang , X. Duan , S. Wang , Environ. Sci. Technol. 2019, 53, 14595. 31721570 10.1021/acs.est.9b05475

[49] H. Zhang , X. Gan , S. Chen , H. Yu , X. Quan , Sep. Purif. Technol. 2022, 292, 121048.

[50] F. Li , P. Wang , T. Zhang , M. Li , S. Yue , S. Zhan , Y. Li , Angew. Chem., Int. Ed. 2023, 62, 202313298. 10.1002/anie.202313298 37795962

[51] Z. Zhao , M. Hu , T. Nie , W. Zhou , B. Pan , B. Xing , L. Zhu , Environ. Sci. Technol. 2023, 57, 4556. 36894515 10.1021/acs.est.2c09336

[52] J. Wang , Q. Fu , J. Yu , H. Yang , Z. Hao , F. Zhu , G. Ouyang , Proc. Natl. Acad. Sci. USA 2022, 119, 2114138119. 10.1073/pnas.2114138119 PMC8784125 35017300

[53] X. Zhou , M.‐K. Ke , G.‐X. Huang , C. Chen , W. Chen , K. Liang , Y. Qu , J. Yang , Y. Wang , F. Li , H.‐Q. Yu , Y. Wu , Proc. Natl. Acad. Sci. USA 2022, 119, 2119492119. 10.1073/pnas.2119492119 PMC8872710 35165185

[54] Y. Z. Peng , G. C. Guo , S. Guo , L. H. Kong , T. B. Lu , Z. M. Zhang , Angew. Chem., Int. Ed. 2021, 60, 22062. 10.1002/anie.202109968 34342372

[55] J. Zhen , J. Sun , X. Xu , Z. Wu , W. Song , Y. Ying , S. Liang , L. Miao , J. Cao , W. Lv , C. Song , Y. Yao , M. Xing , Angew. Chem., Int. Ed. 2024, 63, 202402669. 10.1002/anie.202402669 38637296

[56] Z. W. Wang , E. Almatrafi , H. Wang , H. Qin , W. J. Wang , L. Du , S. Chen , G. M. Zeng , P. Xu , Angew. Chem. Int. Ed. 2022, 61, e202202338. 10.1002/anie.202202338 35514041

[57] M. Kohantorabi , G. Moussavi , S. Giannakis , Chem. Eng. J. 2021, 411, 127957. 10.1016/j.chemosphere.2021.130271 33770697

