# Supplementary material for: Regulating Spin Polarization through Topological Defects in Carbon‐Based Metal‐Free Catalyst for Enhanced Fenton‐Like Activity
Source: Adv Sci (Weinh). 2025 Aug 26;12(42):e14429. doi: 10.1002/advs.202514429 (PMC12622554; doi:10.1002/advs.202514429)
Supplement: Supplementary file 1 — Supporting Information [file ADVS-12-e14429-s001.docx]

Regulating spin polarization through topological defects in carbon-based metal-free catalyst for enhanced Fenton-like activity

Huajie Zhong^a^, Zeyu Gong^a^, Xi Chen^a^, Bin Zhang^b^, Tao Zhan^a^, Jiaxing Yu^c^, Yu Hou^d^, Yuan Tao^b^, Qi Fu^b^, Huangsheng Yang^b^, Jiating Zhen^a^, Duochu Su^e^, Ganggang Li^f^, Junhui Wang^a,^*, Gangfeng Ouyang^a,c,^*

^a^ School of Chemical Engineering and Technology, Sun Yat-Sen University, Zhuhai Guangdong 519082, P. R. China

^b^ School of Biology and Biological Engineering, South China University of Technology, Guangzhou Guangdong, 510006, P. R. China

^c.^ MOE Key Laboratory of Bioinorganic and Synthetic Chemistry/KLGHEI of Environment and Energy Chemistry, School of Chemistry, Sun Yat-Sen University, Guangzhou, Guangdong, 510275, P. R. China

^d^ Guangzhou Customs Technology Center, Guangzhou, Guangdong 510623, China

^e^ Foshan Fosun Chancheng Hospital, Foshan, Guangdong 528000, P. R. China

^f^ National Engineering Laboratory for VOCs pollution Control Material & Technology, Research Center for Environmental Material and Pollution Control Technology, University of Chinese Academy of Sciences, Beijing, 101408, P.R. China

* Corresponding author:

Tel: +86 020 84110845/0953; fax: +86 020 84110845/0953.

E-mail address: cesoygf@mail.sysu.edu.cn (Gangfeng Ouyang)

Characterizations

Raman spectra were recorded on a Renishaw InVia spectrometer with a model 100 Ramascope optical fiber instrument. X-ray photoelectron spectroscopic (XPS) analysis was conducted on an ESCALAB Xi+ spectrometer (Thermo Fisher Scientific Corporation, USA) with Al Kα radiation as the exciting source (250 W). The binding energies of the recorded XPS spectra were corrected according to the C 1s line at 284.8 eV. The pentagons were inspected using a high-resolution transmission electron microscope (JEM-2010HR, JEOL, Japan). The electron paramagnetic resonance spectroscopy measurements (EPR) were obtained on a JESFA-200 (JEOL, Japan) spectrometer. Brunauer-Emmett-Teller (BET) surface areas were investigated bt nitrogen adsorption and desrption at liquid nitrogen temperature (77K) using a volumetric adsorption analyzer (JW-BK200C, JWGB, China).

Density Functional Theory (DFT) Calculations:

All the calculations are performed in the framework of the density functional theory with the projector augmented plane-wave method, as implemented in the Vienna ab initio simulation package ^[1]^. The generalized gradient approximation proposed by Perdew, Burke, and Ernzerhof is selected for the exchange-correlation potential ^[2]^. Weak van der Waals interaction is considered by the DFT-D3 functional ^[3]^. The cutoff energy for the plane wave is set to 400 eV. The energy criterion is set to 10^−5^ eV in the iterative solution of the Kohn−Sham equation. The Brillouin zone integration is performed at the Gamma point. All the structures are relaxed until the residual forces on the atoms have declined to less than 0.05 eV/Å. In this work, single wall carbon nanotubes are taken as an example to consider the catalytic effect for persulfate adsorption and activation. The adsorption energy is studied by DFT calculations, defined as

E_ads_= E_total_ − E_substrate_ - E_molecule_

Where E_total_, E_substrate_, and E_molecule_ denote the total energy of substrate with adsorbate, substrate, and free molecule, respectively.

The Gibbs free energy is defined as

G=E_tot_+E_ZPE_-TS

where E_tot_, E_ZPE_, and TS are total energy, zero-point energy, and entropy of the system.

Catalytic Activity Measurements

The experiments were carried out in a beaker at room temperature. The pH value of the entire reaction system was not further adjusted by any buffers. The reaction solution was prepared by suspending catalysts in 10 ppm 4-CP solution (C_0_=0.08 mM). After reaching the adsorption equilibrium, the reaction was initiated by adding prepared solution of PMS (150 mM). Then samples were withdrawn using a 1 ml syringe at certain intervals during the 60 minutes reaction, and the solid was removed by filtration (0.22μm PTEF filter). The concentration of 4-CP was analyzed using a HPLC (Shimadzu LC-20AD). Separation was performed on a Poroshell 120 EC-C18 column (4.6×100 mm, 2.7μm, Agilent Technology, USA) using a mobile phase consisting of a binary mixture of water and methanol at a flow rate of 0.16mL/min and 0.48mL/min individually. The concentration of PMS was measured by the ABTS colorimetric method. The ABTS colorimetric method is the concentration of PMS was determined based on a previously proposed 2,2’-azino-bis (3-ethylbenzothiazoline-6-sulfonic acid) diammonium salt (ABTS) colorimetric method. The detailed experimental procedures are as follows. Briefly, 0.5 mL filtered sample was added into a 10 mL colorimetric tube, followed by successive addition of 1 mL pH=4 acetic buffer solution, 20 μL KI solution (1.5mM), and 1 mL ABTS solution (2mM), and then was diluted with ultrapure water. The spectrophotometry was performed at a detection wavelength of 415nm.


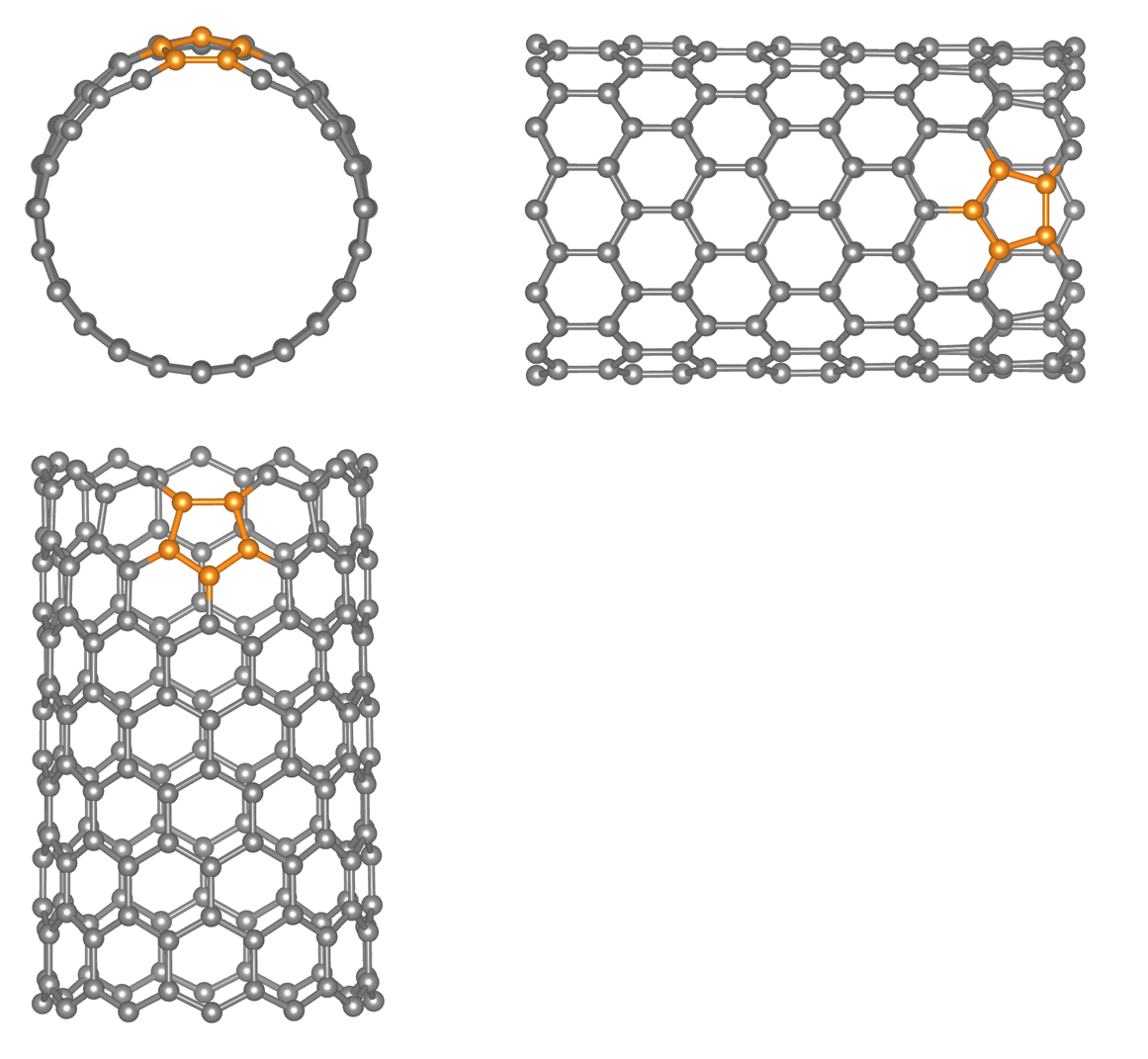


**Figure S1.** The structure of E-C5 from different views.


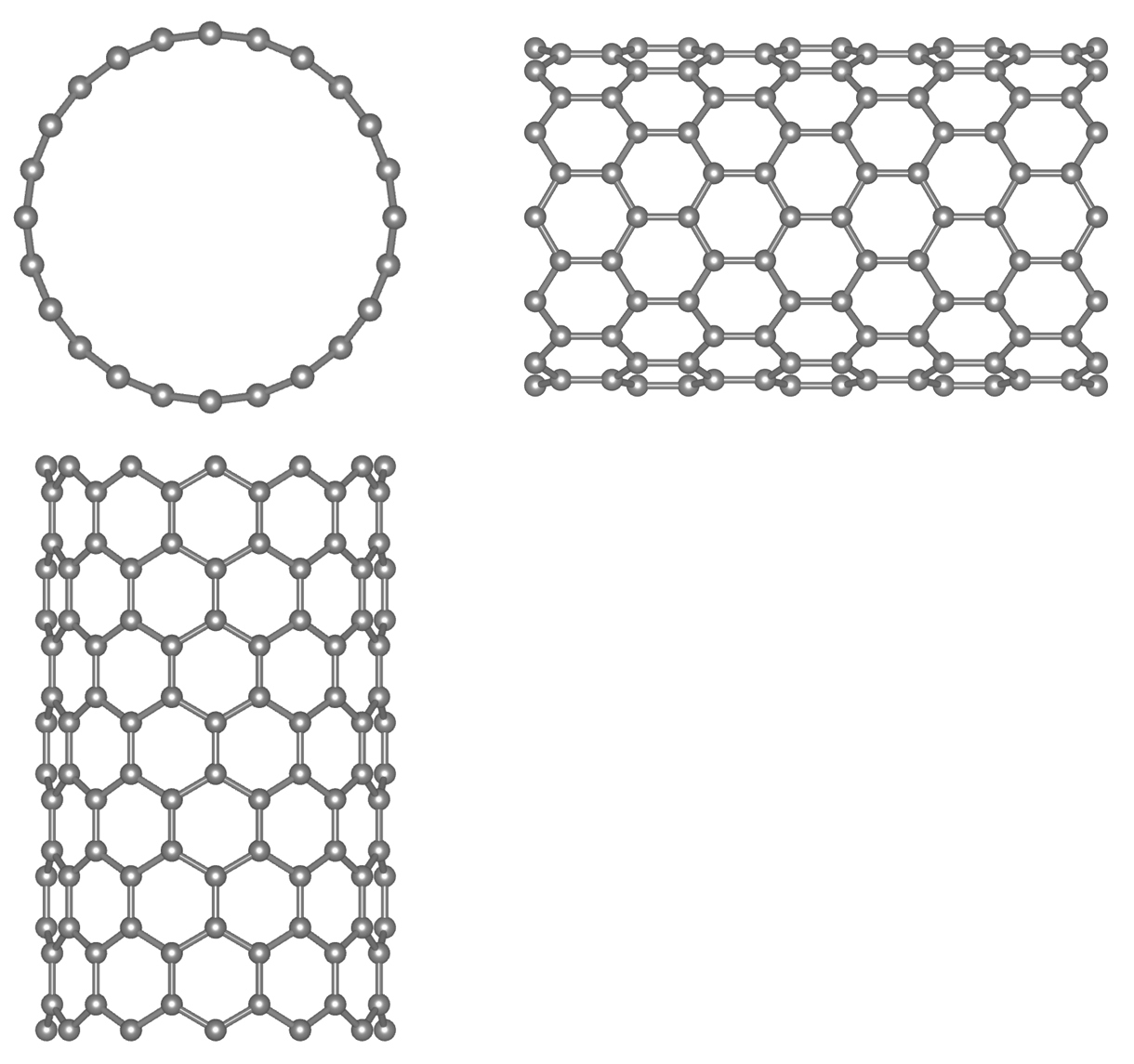


**Figure S2.** The structure of E-C6 from different views.


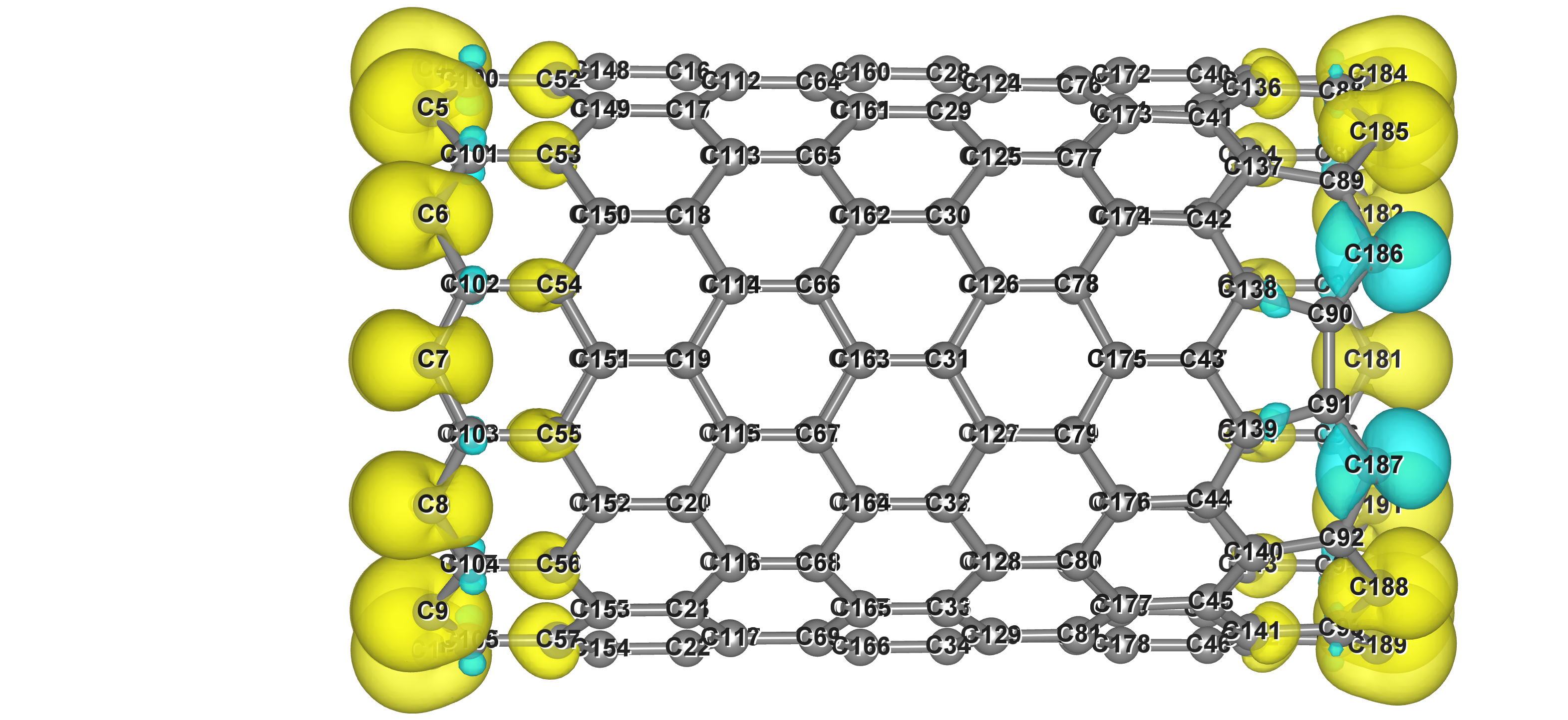


**Figure S3.** Spin density distributions of E-C5.


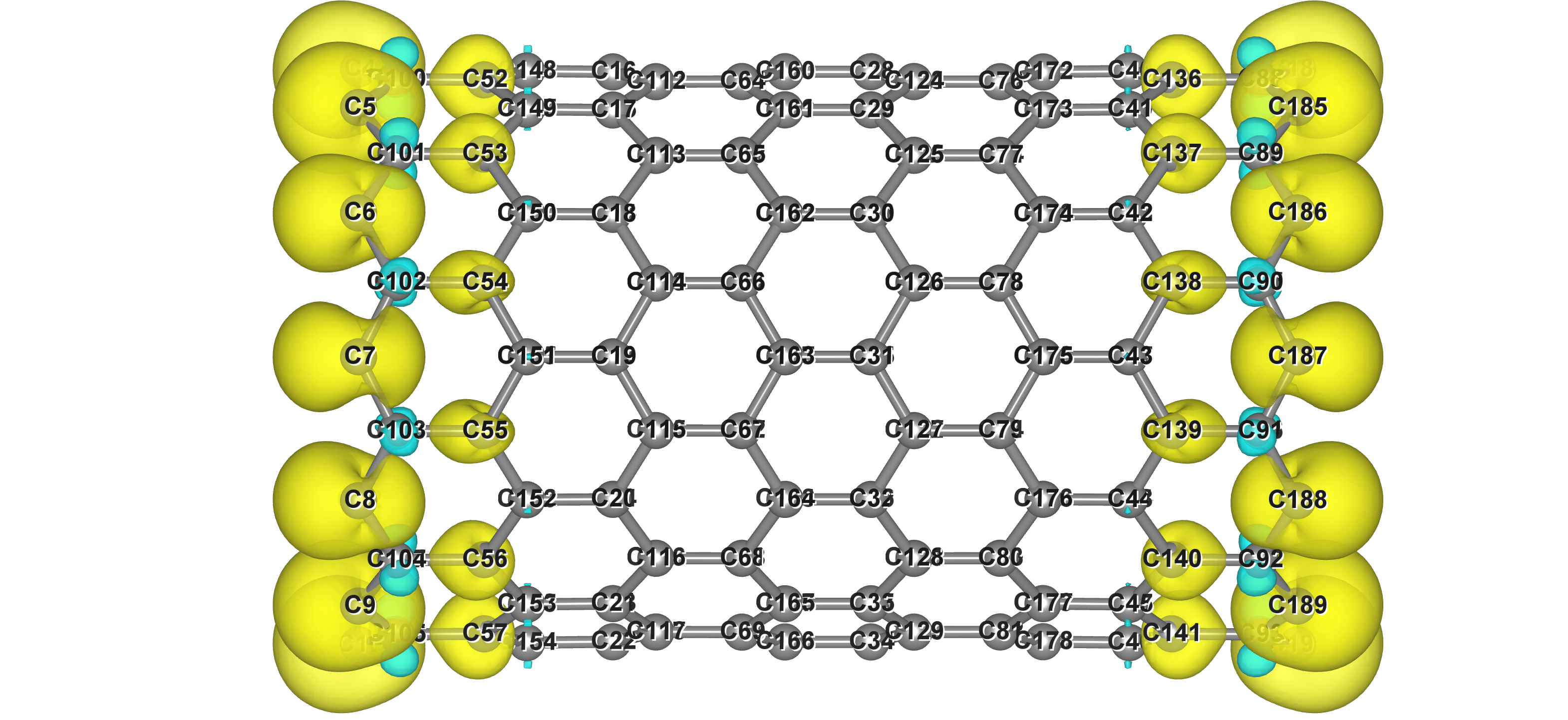


**Figure S4.** Spin density distributions of E-C6.


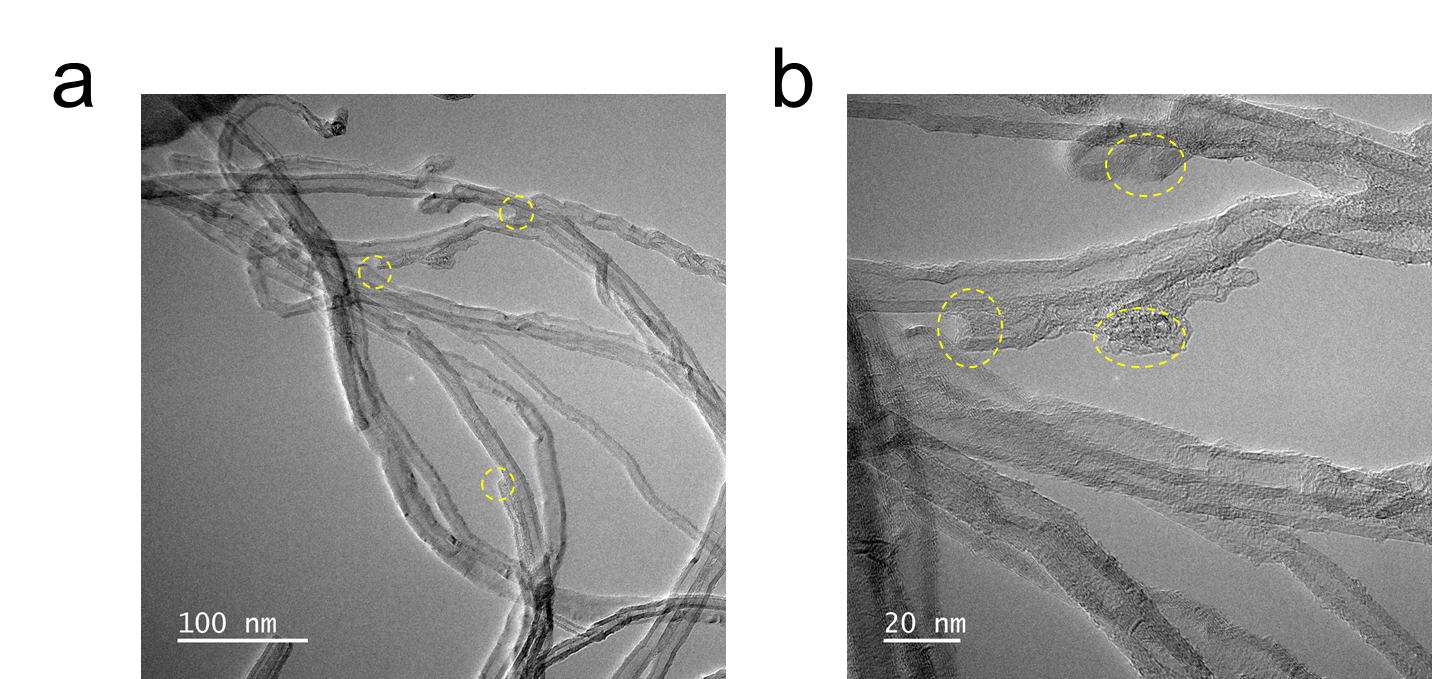
**Figure S5.** TEM images of E-C6

The fractured structure was also observed in the TEM images of E-C6, suggesting the edge defects was produced in E-C6


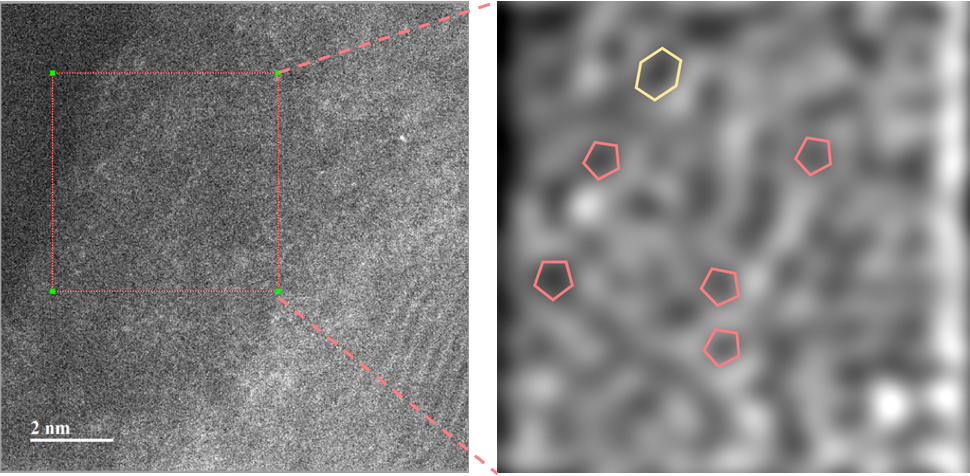


**Figure S6.** AC-STEM image of E-C5 and the corresponding Fourier transform fitting of the selected area.

**Figure S7.** XRD spectra of CNT, E-C5 and E-C6.

Figure S8. Raman spectra of E-C5, E-C6 and CNT.


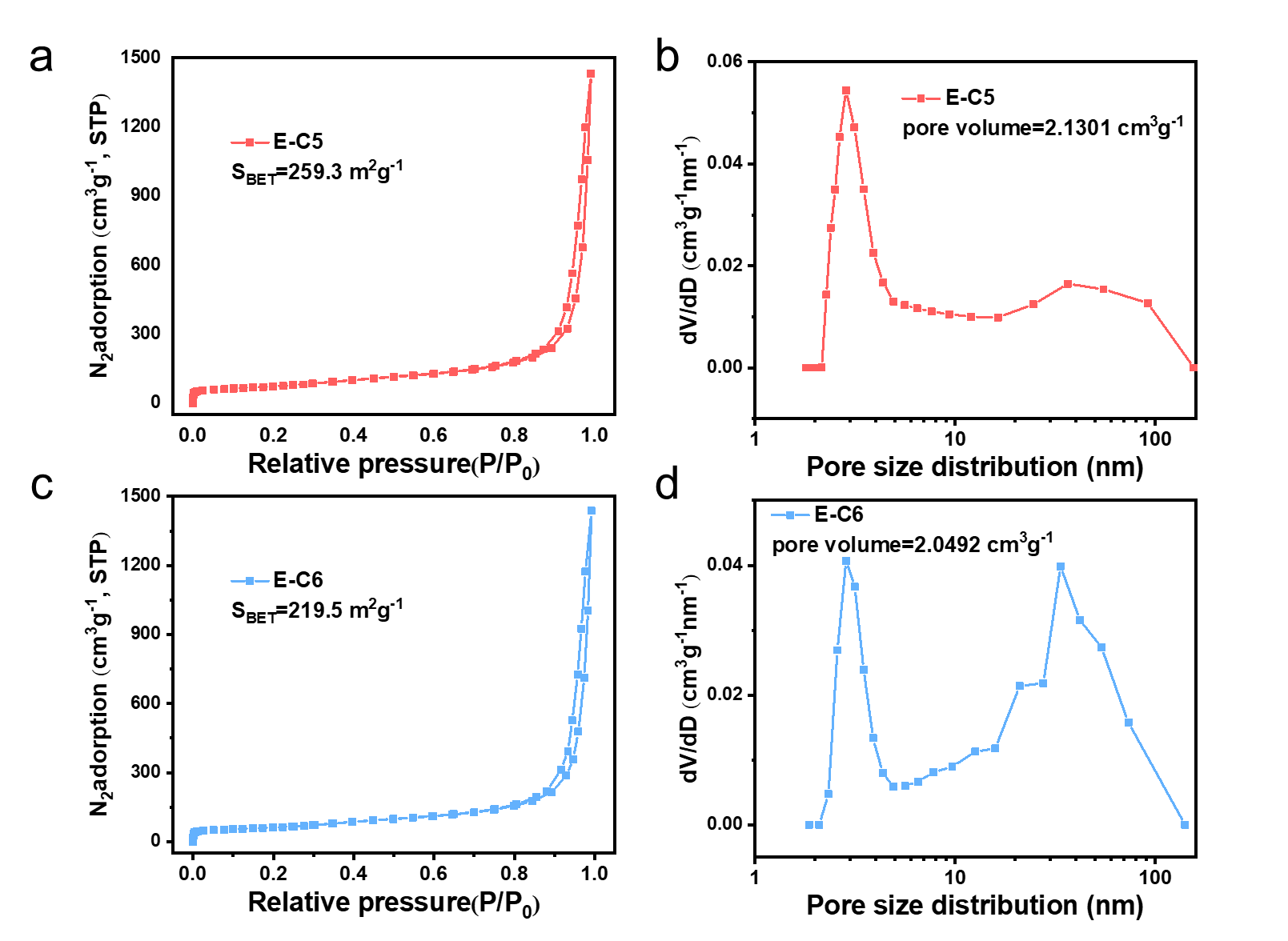


**Figure S9.** (a, c) N_2_ adsorption and desorption isotherms of E-C5 and E-C6; (b, d) the corresponding pore-size distribution curves of E-C5 and E-C6.

**Figure S10.** XPS spectra of CNT, E-C5 and E-C6.

According to the XPS spectra of CNT, E-C5 and E-C6, it can be referred that the CNT was extremely purified. Thus, combined with the Raman spectra, it is an optimized model catalyst to investigated the catalytic activity induced by the active sites.

**Figure S11.** 4-CP adsorption curves of E-C5 and E-C6. Reaction condition: [4-CP] = 10 mg/L, [catalyst] = 0.1 g/L.

**Figure S12.** Catalytic activities of pristine CNT by PMS activation for 4-CP removal. Reaction condition: [4-CP] = 10 mg/L, [PMS] = 1.5 mM, [catalyst] = 0.1 g/L.

**Figure S13.** Catalytic activities of E-C6-1100 by PMS activation for 4-CP removal. Reaction condition: [4-CP] = 10 mg/L, [PMS] = 1.5 mM, [catalyst] = 0.1 g/L.

**Figure S14.** The kinetic analysis of 4-CP degradation by the pentagon-defects/PMS systems according to pseudo-first-order model.

**Figure S15.** The kinetic analysis of PMS consumption by the pentagon-defects/PMS systems according to pseudo-first-order model.

**Figure S16.** In situ Ramna spectra of E-C5/PMS system over time.


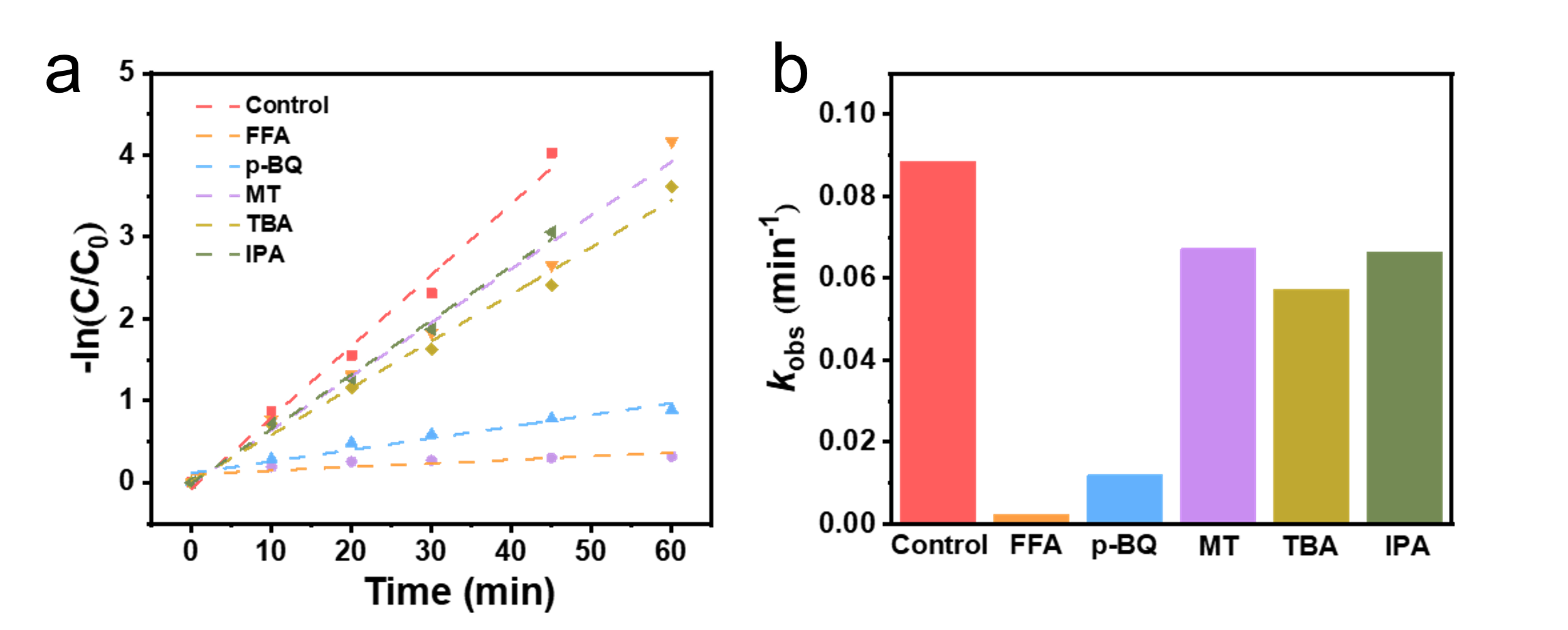


**Figure S17.** (a)The kinetic analysis of quenching test according to pseudo-first-order model and (b) *k_obs_* of E-C5/PMS system under different quenching agents.


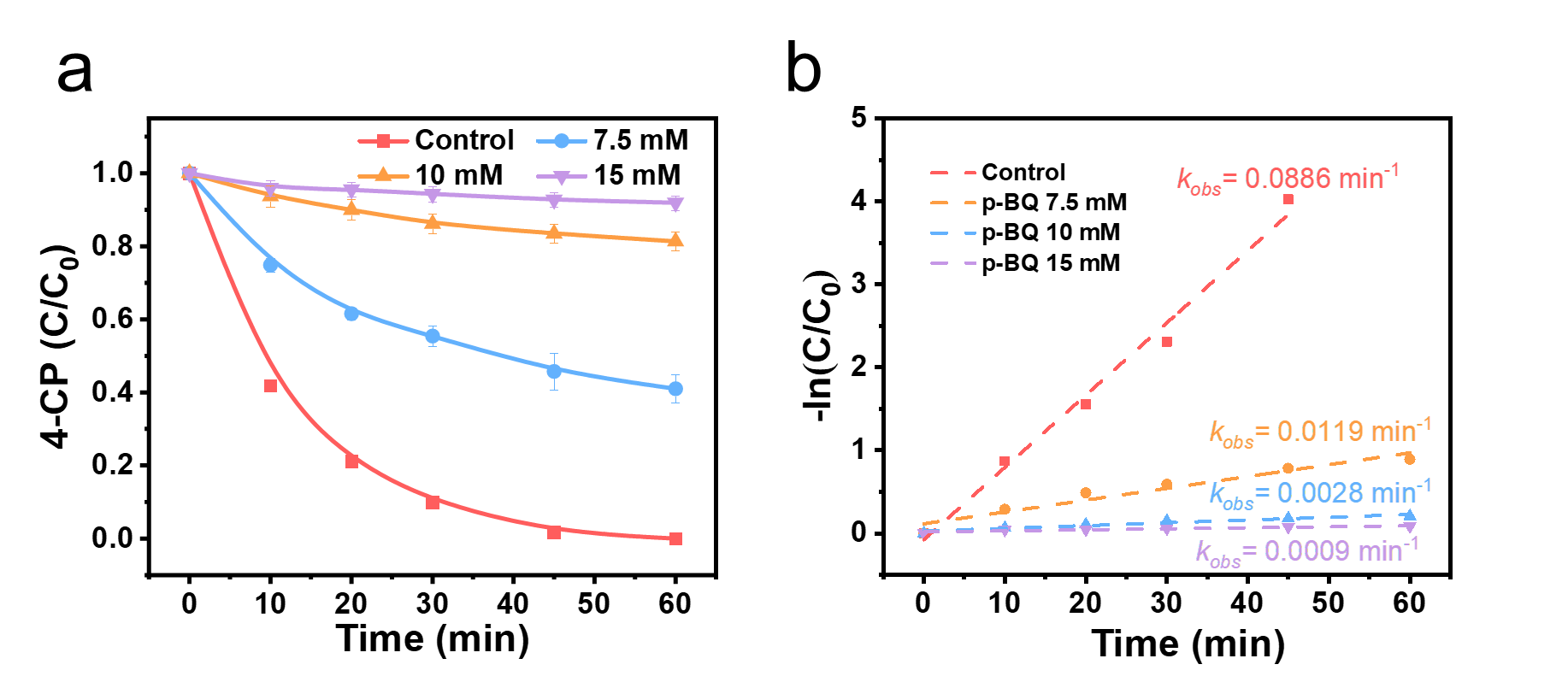


**Figure S18.** (a) 4-CP degradation curves of E-C5/PMS system under different concentration of p-BQ and (b) the corresponding kinetic analysis according to pseudo-first-order model. Reaction condition: [4-CP] = 10 mg/L, [PMS] = 1.5 mM, [catalyst] = 0.1 g/L.

**Figure S19.** EPR spectra obtained by spin trapping with DMPO in water.

**Figure S20.** Production of ^1^O_2_ by E-C5/PMS system and E-C6/PMS system.

**Figure S21.** EPR spectra obtained by spin trapping with DMPO (methanol as the solvent).

**Figure S22.** 4-CP degradation curves of E-C5/PMS system under different pH value. Reaction condition: [4-CP] = 10 mg/L, [PMS] = 1.5 mM, [catalyst] = 0.1 g/L.

**Figure S23.** 4-CP degradation curves of ClO⁻/PMS system under various inorganic anions. Reaction condition: [Cl^-^] = [SO_4_^2-^] = [HCO_3_^-^] = 10 mM, [4-CP] = 10 mg/L, [ClO⁻] = [PMS] = 1.5 mM, pH = 10.

**Figure S24.** 4-CP removal efficiency of E-C5, used E-C5 and regenerated E-N-CNT. Reaction conditions: catalyst dosage = 0.1 g L^-1^, [PMS] = 1.5 mM, [4-CP] = 10 ppm, reaction time = 60 min.


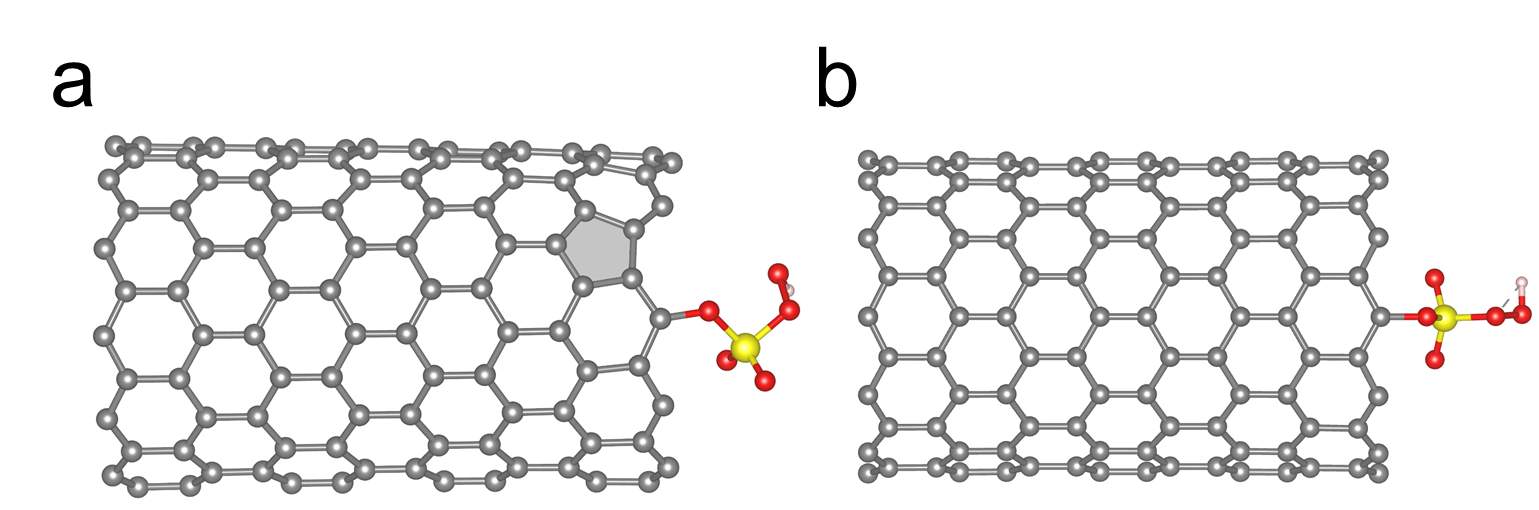


**Figure S25.** The PMS adsorption models of (a) E-C5, (b) E-C6.

**Figure S26.** In situ Raman spectra of E-C5 alone, and E-C5 combined with PMS.

As shown in Figure S13, the bule shift (11.3cm^-1^) was detected after the adsorption of PMS in E-C5, implying PMS sever as an electron accepter attracting electrons from the carbon π states. Furthermore, the red shift of the O-O bond in PMS (Figure S11) confirmed the direction of electron from E-C5 to PMS.


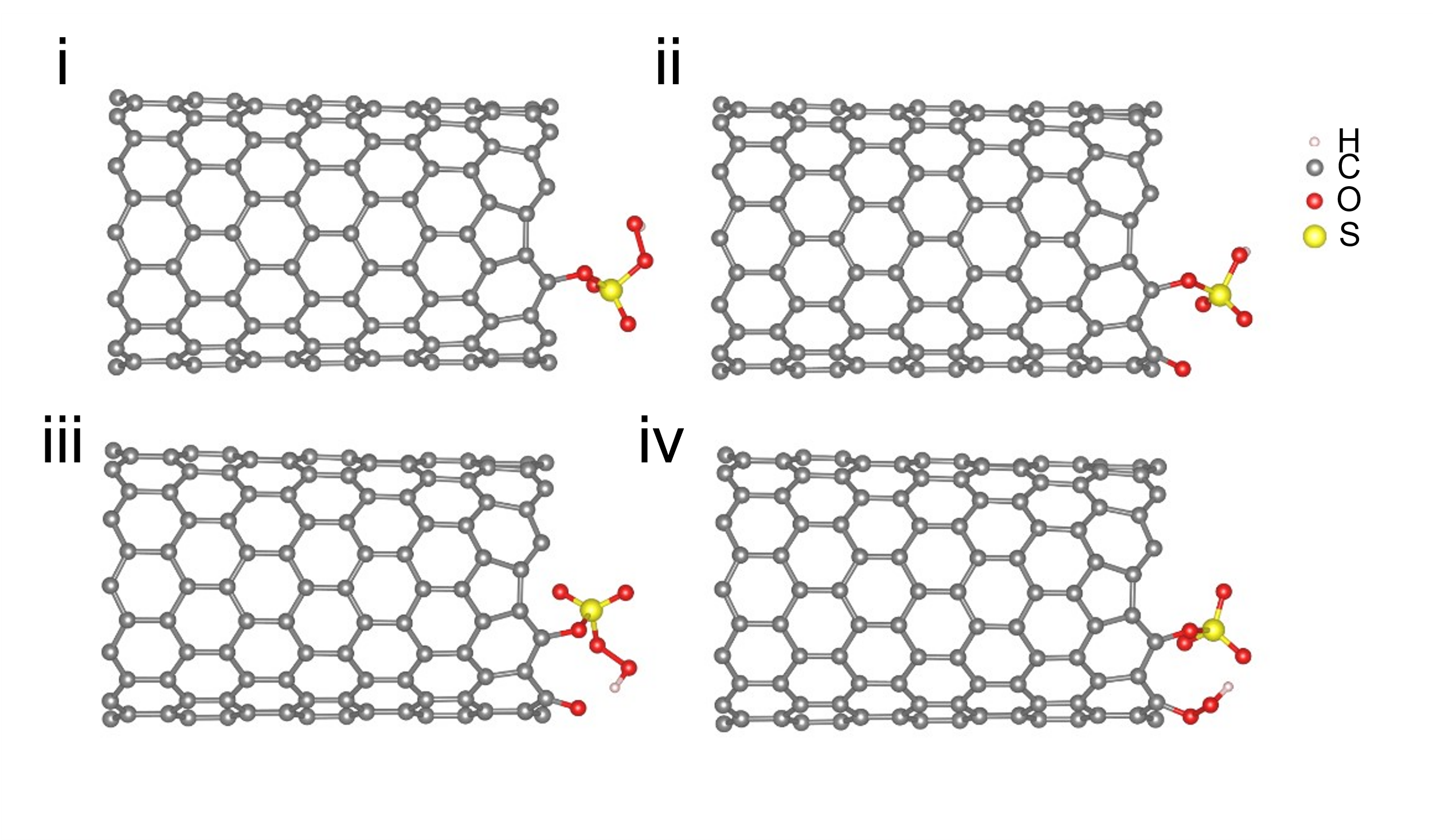


**Figure S27.** The corresponding structures of reaction intermediates for E-C5.


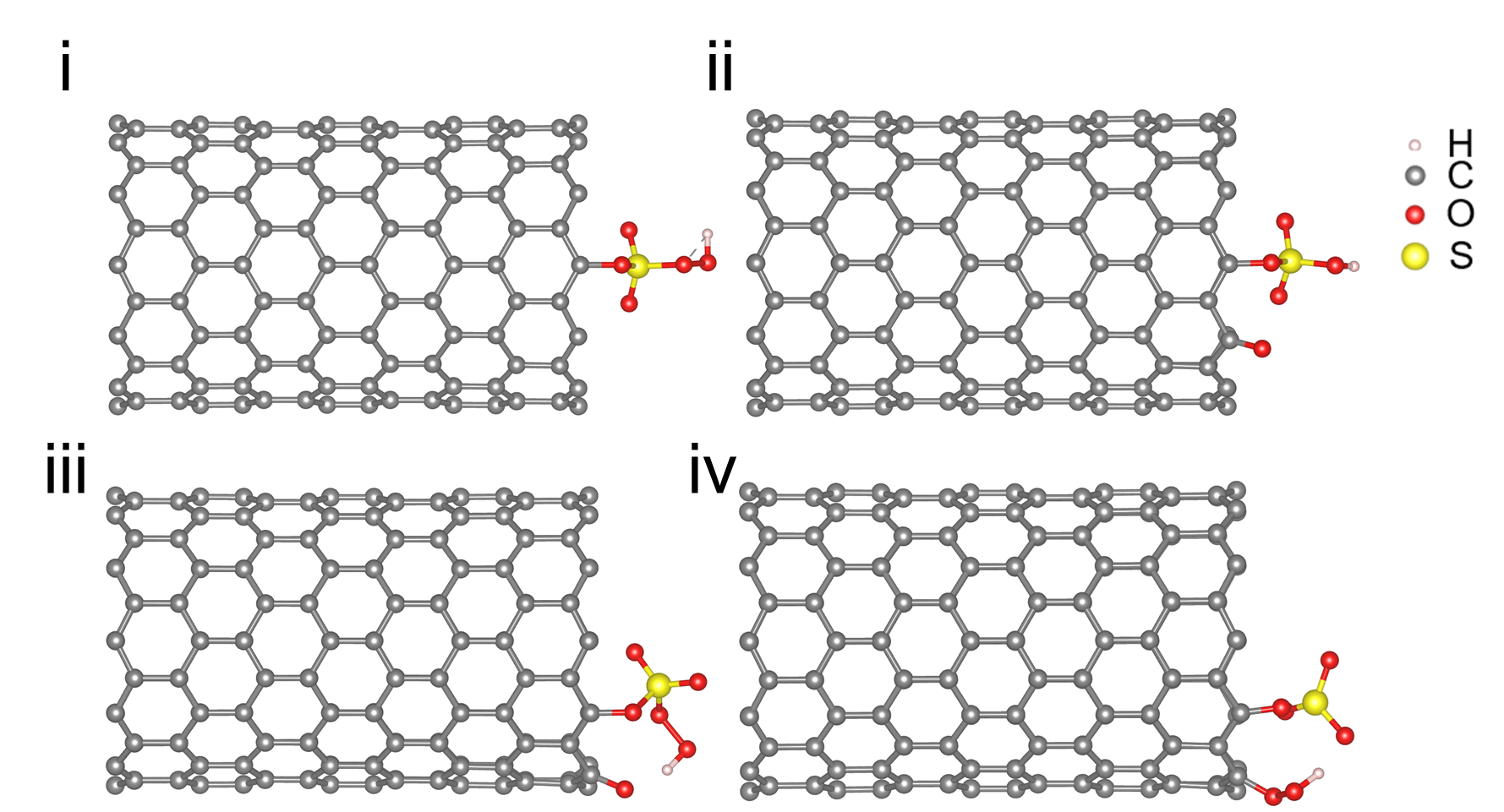


**Figure S28.** The corresponding structures of reaction intermediates for E-C6.

**Table S1.** Spin density of E-C5 for corresponding positions

| Atom | Spin density | Atom | Spin density | Atom | Spin density | Atom | Spin density | Atom | Spin density | Atom | Spin density |
| --- | --- | --- | --- | --- | --- | --- | --- | --- | --- | --- | --- |
| 1 | 0.643 | 17 | 0.019 | 33 | -0.002 | 49 | 0.11 | 65 | 0.003 | 81 | -0.007 |
| 2 | 0.643 | 18 | 0.02 | 34 | -0.002 | 50 | 0.109 | 66 | 0.005 | 82 | -0.008 |
| 3 | 0.643 | 19 | 0.019 | 35 | -0.002 | 51 | 0.11 | 67 | 0.005 | 83 | -0.008 |
| 4 | 0.642 | 20 | 0.02 | 36 | -0.003 | 52 | 0.11 | 68 | 0.003 | 84 | -0.008 |
| 5 | 0.643 | 21 | 0.019 | 37 | -0.017 | 53 | 0.11 | 69 | 0.004 | 85 | -0.032 |
| 6 | 0.644 | 22 | 0.019 | 38 | -0.015 | 54 | 0.111 | 70 | 0.003 | 86 | -0.031 |
| 7 | 0.643 | 23 | 0.019 | 39 | -0.012 | 55 | 0.111 | 71 | 0.002 | 87 | -0.026 |
| 8 | 0.644 | 24 | 0.019 | 40 | -0.01 | 56 | 0.11 | 72 | 0.003 | 88 | -0.016 |
| 9 | 0.643 | 25 | -0.003 | 41 | -0.018 | 57 | 0.11 | 73 | -0.008 | 89 | -0.019 |
| 10 | 0.642 | 26 | -0.003 | 42 | 0.004 | 58 | 0.11 | 74 | -0.008 | 90 | -0.025 |
| 11 | 0.643 | 27 | -0.002 | 43 | -0.02 | 59 | 0.109 | 75 | -0.008 | 91 | -0.025 |
| 12 | 0.643 | 28 | -0.002 | 44 | 0.004 | 60 | 0.11 | 76 | -0.007 | 92 | -0.019 |
| 13 | 0.019 | 29 | -0.002 | 45 | -0.018 | 61 | 0.003 | 77 | -0.004 | 93 | -0.016 |
| 14 | 0.019 | 30 | 0.002 | 46 | -0.01 | 62 | 0.002 | 78 | -0.002 | 94 | -0.026 |
| 15 | 0.019 | 31 | -0.001 | 47 | -0.012 | 63 | 0.003 | 79 | -0.002 | 95 | -0.031 |
| 16 | 0.019 | 32 | 0.002 | 48 | -0.015 | 64 | 0.004 | 80 | -0.004 | 96 | -0.032 |
|  |  |  |  |  |  |  |  |  |  |  |  |
| 97 | -0.032 | 113 | -0.011 | 129 | -0.001 | 145 | -0.017 | 161 | -0.006 | 177 | 0.009 |
| 98 | -0.032 | 114 | -0.011 | 130 | 0 | 146 | -0.017 | 162 | -0.006 | 178 | 0.016 |
| 99 | -0.032 | 115 | -0.011 | 131 | 0.001 | 147 | -0.018 | 163 | -0.005 | 179 | 0.015 |
| 100 | -0.032 | 116 | -0.011 | 132 | 0.001 | 148 | -0.018 | 164 | -0.006 | 180 | 0.013 |
| 101 | -0.032 | 117 | -0.011 | 133 | 0.112 | 149 | -0.018 | 165 | -0.006 | 181 | 0.646 |
| 102 | -0.032 | 118 | -0.011 | 134 | 0.102 | 150 | -0.018 | 166 | -0.005 | 182 | 0.647 |
| 103 | -0.032 | 119 | -0.011 | 135 | 0.09 | 151 | -0.017 | 167 | -0.005 | 183 | 0.644 |
| 104 | -0.032 | 120 | -0.011 | 136 | 0.084 | 152 | -0.018 | 168 | -0.005 | 184 | 0.613 |
| 105 | -0.032 | 121 | 0.001 | 137 | 0.031 | 153 | -0.018 | 169 | 0.013 | 185 | 0.533 |
| 106 | -0.032 | 122 | 0.001 | 138 | -0.014 | 154 | -0.018 | 170 | 0.013 | 186 | -0.438 |
| 107 | -0.032 | 123 | 0 | 139 | -0.014 | 155 | -0.018 | 171 | 0.015 | 187 | -0.438 |
| 108 | -0.032 | 124 | -0.001 | 140 | 0.031 | 156 | -0.017 | 172 | 0.016 | 188 | 0.533 |
| 109 | -0.011 | 125 | -0.001 | 141 | 0.084 | 157 | -0.005 | 173 | 0.009 | 189 | 0.613 |
| 110 | -0.011 | 126 | -0.003 | 142 | 0.09 | 158 | -0.005 | 174 | 0.004 | 190 | 0.644 |
| 111 | -0.011 | 127 | -0.003 | 143 | 0.102 | 159 | -0.005 | 175 | -0.001 | 191 | 0.647 |
| 112 | -0.011 | 128 | -0.001 | 144 | 0.112 | 160 | -0.005 | 176 | 0.004 |  |  |

| Atom | Spin density | Atom | Spin density | Atom | Spin density | Atom | Spin density | Atom | Spin density | Atom | Spin density |
| --- | --- | --- | --- | --- | --- | --- | --- | --- | --- | --- | --- |
| 1 | 0.643 | 17 | 0.018 | 33 | -0.004 | 49 | 0.109 | 65 | 0.002 | 81 | -0.01 |
| 2 | 0.643 | 18 | 0.018 | 34 | -0.004 | 50 | 0.109 | 66 | 0.002 | 82 | -0.01 |
| 3 | 0.643 | 19 | 0.018 | 35 | -0.004 | 51 | 0.109 | 67 | 0.002 | 83 | -0.01 |
| 4 | 0.643 | 20 | 0.018 | 36 | -0.004 | 52 | 0.109 | 68 | 0.002 | 84 | -0.01 |
| 5 | 0.643 | 21 | 0.018 | 37 | -0.017 | 53 | 0.109 | 69 | 0.002 | 85 | -0.032 |
| 6 | 0.643 | 22 | 0.018 | 38 | -0.017 | 54 | 0.109 | 70 | 0.002 | 86 | -0.032 |
| 7 | 0.643 | 23 | 0.018 | 39 | -0.017 | 55 | 0.109 | 71 | 0.002 | 87 | -0.032 |
| 8 | 0.643 | 24 | 0.018 | 40 | -0.017 | 56 | 0.109 | 72 | 0.002 | 88 | -0.032 |
| 9 | 0.643 | 25 | -0.004 | 41 | -0.017 | 57 | 0.109 | 73 | -0.01 | 89 | -0.032 |
| 10 | 0.643 | 26 | -0.004 | 42 | -0.017 | 58 | 0.109 | 74 | -0.01 | 90 | -0.032 |
| 11 | 0.643 | 27 | -0.004 | 43 | -0.017 | 59 | 0.109 | 75 | -0.01 | 91 | -0.032 |
| 12 | 0.643 | 28 | -0.004 | 44 | -0.017 | 60 | 0.109 | 76 | -0.01 | 92 | -0.032 |
| 13 | 0.018 | 29 | -0.004 | 45 | -0.017 | 61 | 0.002 | 77 | -0.01 | 93 | -0.032 |
| 14 | 0.018 | 30 | -0.004 | 46 | -0.017 | 62 | 0.002 | 78 | -0.01 | 94 | -0.032 |
| 15 | 0.018 | 31 | -0.004 | 47 | -0.017 | 63 | 0.002 | 79 | -0.01 | 95 | -0.032 |
| 16 | 0.018 | 32 | -0.004 | 48 | -0.017 | 64 | 0.002 | 80 | -0.01 | 96 | -0.032 |
|  |  |  |  |  |  |  |  |  |  |  |  |
| 97 | -0.032 | 113 | -0.01 | 129 | 0.002 | 145 | -0.017 | 161 | -0.004 | 177 | 0.018 |
| 98 | -0.032 | 114 | -0.01 | 130 | 0.002 | 146 | -0.017 | 162 | -0.004 | 178 | 0.018 |
| 99 | -0.032 | 115 | -0.01 | 131 | 0.002 | 147 | -0.017 | 163 | -0.004 | 179 | 0.018 |
| 100 | -0.032 | 116 | -0.01 | 132 | 0.002 | 148 | -0.017 | 164 | -0.004 | 180 | 0.018 |
| 101 | -0.032 | 117 | -0.01 | 133 | 0.109 | 149 | -0.017 | 165 | -0.004 | 181 | 0.643 |
| 102 | -0.032 | 118 | -0.01 | 134 | 0.109 | 150 | -0.017 | 166 | -0.004 | 182 | 0.643 |
| 103 | -0.032 | 119 | -0.01 | 135 | 0.109 | 151 | -0.017 | 167 | -0.004 | 183 | 0.643 |
| 104 | -0.032 | 120 | -0.01 | 136 | 0.109 | 152 | -0.017 | 168 | -0.004 | 184 | 0.643 |
| 105 | -0.032 | 121 | 0.002 | 137 | 0.109 | 153 | -0.017 | 169 | 0.018 | 185 | 0.643 |
| 106 | -0.032 | 122 | 0.002 | 138 | 0.109 | 154 | -0.017 | 170 | 0.018 | 186 | 0.643 |
| 107 | -0.032 | 123 | 0.002 | 139 | 0.109 | 155 | -0.017 | 171 | 0.018 | 187 | 0.643 |
| 108 | -0.032 | 124 | 0.002 | 140 | 0.109 | 156 | -0.017 | 172 | 0.018 | 188 | 0.643 |
| 109 | -0.01 | 125 | 0.002 | 141 | 0.109 | 157 | -0.004 | 173 | 0.018 | 189 | 0.643 |
| 110 | -0.01 | 126 | 0.002 | 142 | 0.109 | 158 | -0.004 | 174 | 0.018 | 190 | 0.643 |
| 111 | -0.01 | 127 | 0.002 | 143 | 0.109 | 159 | -0.004 | 175 | 0.018 | 191 | 0.643 |
| 112 | -0.01 | 128 | 0.002 | 144 | 0.109 | 160 | -0.004 | 176 | 0.018 | 192 | 0.643 |

**Table S2.** Spin density of E-C6 for corresponding positions

**Table S3**. The HPLC analysis conditions for different substrates.

| Substrates | Mobile phase | Ratio  (v:v) | Wavelength (nm) | Flow  (mL min^-1^) |
| --- | --- | --- | --- | --- |
| 4-CP | methanol/water | 70:30 | 281 | 0.6 |
| BPA | methanol/water | 70:30 | 226 | 0.6 |
| RhB | methanol/water | 70:30 | 554 | 0.6 |
| Phenol | methanol/water | 50:50 | 271 | 0.6 |
| SD | acetonitrile/water | 70:30 | 265 | 0.6 |

**Table S4.** XPS analyses of different catalysts.

| Sample | C/At.% | O/At.% | Pyridinic N/At.% | Pyrrolic N/At.% | Graphitic N/At.% |
| --- | --- | --- | --- | --- | --- |
| CNT | 99.78 | 0.22 | / | / | / |
| E-N-CNT | 99.01 | 0.73 | 0.18 | 0.08 | / |
| E-C6 | 99.47 | 0.53 | / | / | / |
| E-C5 | 99.24 | 0.76 | / | / | / |

[1] G. Kresse, J. Furthmüller, *Phys. Rev. B* **1996**, *54*, 11169-11186.

[2] J. P. Perdew, K. Burke, M. Ernzerhof, *Phys. Rev. Lett.* **1996**, *77*, 3865-3868.

[3] S. Grimme, J. Antony, S. Ehrlich, H. Krieg, *J. Chem. Phys.* **2010**, *132*, 154104.
